# Agglomerations of Methane Hydrate Particles in Aqueous Solutions: Insight from Dissipative Particle Dynamics Simulations

**DOI:** 10.1021/acsomega.5c10009

**Published:** 2026-03-05

**Authors:** Minglei Wang, Pinqiang Cao

**Affiliations:** School of Resource and Environmental Engineering, 47900 Wuhan University of Science and Technology, Wuhan, Hubei 430081, China

## Abstract

Despite the common occurrence of hydrate particle agglomerations in engineering applications and naturally occurring environments, there is still a gap in exploring the agglomeration mechanism of gas hydrate particles in aqueous solutions due to the experimental challenges and limitations. Herein, particle agglomerations of methane hydrates are investigated by using dissipative particle dynamics. Our results show that the agglomeration behaviors of methane hydrate particles are related to particle sizes, the particle size ratios, and the shapes of hydrate particles. Before hydrate particle agglomerations, the distance between any two hydrate particles in these hydrate particle systems exhibits an oscillation manner, and their fluctuation amplitude strongly depends on the particle sizes. Furthermore, the hydrate particle motions in those agglomeration processes are consistent with previous studies at the microscopic scale. This work not only extends the research scale of hydrate particle agglomerations but also provides a new computational method framework for gas hydrate communities in the world.

## Graphical abstract

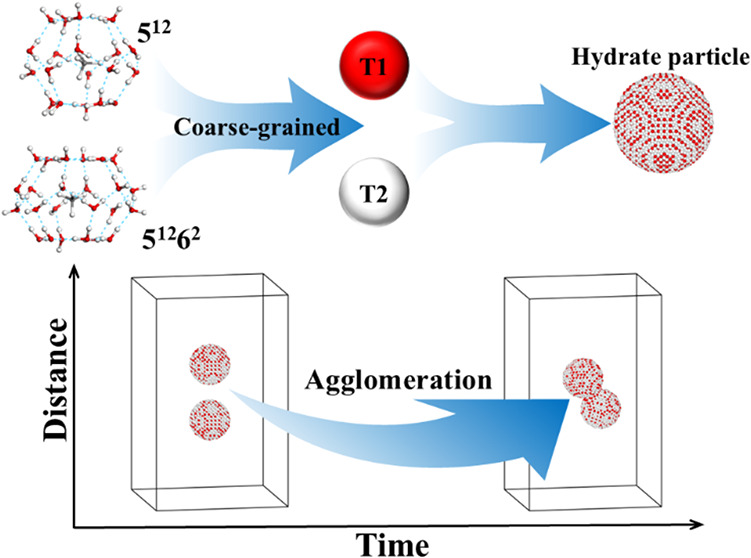

## Introduction

Gas hydrates are crystalline solid substances formed by hydrogen-bond water frameworks and suitable guest gas molecules at low temperatures and high pressures.^1–3^ Up to now, three main types of hydrate crystal structures have been discovered, namely, structure I (sI), structure II (sII), and hexagonal H (sH). Naturally occurring gas hydrates are widely found in permafrost regions and seabed sediments at continental margins.^4,5^ They are considered to be one of the potential energy sources due to their high energy density and enormous reserves.^6–8^ Moreover, some research fields in the hydrate community have attracted a lot of attention, e.g., mechanical properties of gas hydrates,^9–12^ phase stability of gas hydrates,^13–15^ and flow assurance in oil and gas pipelines.^2,3^ Such a challenge in flow assurance and safety issues in oil and gas pipelines can lead to a threat to the engineering industry.^16,17^ To address those issues, some hydrate additives, e.g., thermodynamic inhibitors, kinetic inhibitors, and antiagglomerants have been developed.^16,18–21^ These additives can either fundamentally delay hydrate nucleation and growth or prevent agglomerations of gas hydrates.^22,23^

To date, significant advancements have been made on the adhesion properties of gas hydrate particles^24,25^ and hydrate agglomerations.^26–28^ Moreover, a micromechanical force apparatus (MMF) offers a direct approach for quantitatively understanding agglomeration mechanisms of hydrate particles.^29,30^ By MMF, it is found that the adhesion force between tetrahydrofuran hydrate particles in *n-decane* is directly dependent on the contact time at atmospheric pressure and temperature ranging from 261 to 275 K.^30^ Moreover, the adhesion force among hydrate particles in tetrahydrofuran hydrate/*n*-decane/tetrahydrofuran hydrate systems is also measured at atmospheric pressure over a temperature range of 263–275 K.^31^ At 3.2 °C and atmospheric pressure, the adhesion forces of cyclopentane hydrate are weaker in a cyclopentane bulk fluid containing small amounts of crude oil than in pure cyclopentane.^32^ Under high-pressure MMF, annealing time is a key factor influencing interparticle bonding force.^29^ The adhesion force of CH_4_/C_2_H_6_ hydrates in a high-pressure liquid hydrocarbon system decreases with increasing annealing time but increases with increasing contact time.^33^ Moreover, interfacial properties of gas hydrates are elaborated to understand the interfacial phenomenal mechanism of gas hydrates.^34–36^ By numerical simulation techniques, a computational fluid dynamics (CFD) model is developed to simulate gas hydrate agglomeration, showing close agreement with previous data.^37,38^ Importantly, two reunion mechanisms are discussed: namely, a contact-induced mechanism, which attributes agglomeration to contacts between water droplets and hydrate particles, and a shear-restricted mechanism, dictated by an equilibrium of hydrodynamic and adhesion forces.^26^ Interestingly, by molecular dynamics simulations, it is found that molecular structural evolutions of both hydrate and sand nanoparticles are characterized by translational and rotational motions, and hydrate nanoparticles are finally connected with sand nanoparticles.^39^

Over the past few decades, dissipative particle dynamics (DPD) methods have been widely applied to understand particle aggregation in various particle systems to obtain profound insights. For example, DPD simulations revealed that the size and spatial positioning of aggregates can be controlled by designing the interactions between particle surfaces and environments,^40^ providing a theoretical basis for the controllable design of nanoparticles in template-based assembly. Moreover, DPD has successfully clarified the influence of the inherent structural characteristics of particles or molecules on their self-assembly behavior,^41^ offering a reliable correlation from molecular design to macroscopic morphology. Furthermore, a mesoscale “coarse-grain” model for hydroxypropyl-methylcellulose (HPMC), poly­(vinylpyrrolidone) (PVP), microcrystalline cellulose (MCC), poly­(ethylene glycol) (PEG), and stearic acid (SA) was proposed to describe the structure of colloidal suspensions composed of the polymers by DPD simulations.^42^ These works verified and expanded the capabilities of DPD in those fields from multiple dimensions. Understanding the agglomeration processes of hydrate particles is of great importance in flow assurance and safety issues induced by gas hydrates. However, the agglomeration mechanisms of gas hydrate particles remain unknown at microscopic and mesoscopic scales. This work utilizes dissipative particle dynamics to study the agglomerations of methane hydrate particles in aqueous solutions. The effects of the particle size ratio, particle size ratio, shapes of particles, and particle number on agglomerations of methane hydrate particles are examined. The objective of these DPD simulations is to transcend spatial and temporal scales of microscopic agglomeration processes and to elucidate their agglomeration mechanisms. The novelty of this work is the new idea of the modeling method to simulate agglomeration behaviors of hydrate particles to enhance our multiscale understanding of hydrate particle agglomeration processes. This new coarse-grained scheme for sI methane hydrate can achieve the simplest coarse-grained representation from the molecular composition of hydrate structures. The rigid methane hydrate structure is then simulated by DPD simulations. Moreover, different particle agglomeration patterns are discussed in this work.

## Methodology

### DPD Methods

Dissipative particle dynamics is a mesoscopic simulation method, and it can be used to simulate the behavior of complex fluids. The DPD method was proposed by Hoogerbrugge and Koelman^43^ in 1992 to address the differences between lattice automaton methods and practical applications, as well as fluid problems at mesoscale time and space scales that molecular dynamics (MD) cannot solve.^43,44^ In the DPD method, one bead represents a group of atoms or a volume of fluid that is large on the atomistic scale but small on the macroscopic scale. The motion of each DPD bead in the system is governed by Newton’s equations of motion:

1 $$\frac{dR_{i}}{dt}=v_{i}$$

2 $$\frac{dv_{i}}{dt}=\frac{f_{i}}{m_{i}}$$

where *R*_*i*_, *v*_*i*_, *f*_*i*_, and *m*_*i*_ are displacement, velocity, total force, and mass for the bead *i*, respectively. For simplicity, all units used in this work are reduced DPD units.^45^ Each bead can interact with each other by three nonbonded interactions within a specific cutoff radius (*r*_c_). The total force between two beads of *i* and *j* consists of three parts, e.g., conservative force (*F*_*ij*_^C^), dissipative force (*F*_*ij*_^D^), and random force (*F*_*ij*_^R^), as follows:

3 $$f_{i}=\sum_{j\neq i}\left(F_{\mathrm{ij}}^{C}+F_{\mathrm{ij}}^{D}+F_{\mathrm{ij}}^{R}\right)$$

The conservative force (*F*_*ij*_^C^) is a soft repulsive interaction force. Dissipative (*F*_*ij*_^D^) and random (*F*_*ij*_^R^) forces act as built-in thermostats to maintain the temperature *T* of the system, and the equilibrium temperature *T* satisfies the fluctuating dissipation theorem.^46^ The specific expressions of these three forces are given below:

4 $$F_{\mathrm{ij}}^{C}=a_{\mathrm{ij}}\omega^{C}\left(r\right){\mathrm{r̂}}_{\mathrm{ij}}$$

5 $$F_{\mathrm{ij}}^{D}=-\gamma_{\mathrm{ij}}\omega^{D}\left(r\right)\left(v_{\mathrm{ij}}\cdot {\mathrm{r̂}}_{\mathrm{ij}}\right){\mathrm{r̂}}_{\mathrm{ij}}$$

6 $$F_{\mathrm{ij}}^{R}=\sigma_{\mathrm{ij}}\omega^{R}\left(r\right)\xi_{\mathrm{ij}}\Delta t^{-1/2}{\mathrm{r̂}}_{\mathrm{ij}}$$

where *r*_*ij*_ = *r*_*i*_ – *r*_*j*_, *r̂*_*ij*_ = *r*_*ij*_/|*r*_*ij*_|, and *v*_*ij*_ *= v*_*i*_ *– v*_*j*_, where *r*_*i*_ and *r*_*j*_ are the positions of bead *i* and bead *j*, respectively; *r̂*_*ij*_ is the unit position vector; *v*_*i*_ and *v*_*j*_ are the velocities of bead *i* and bead *j*, respectively; and *a*_*ij*_ is the repulsion parameter between bead *i* and bead *j*. The calculation of the repulsion parameters is one of the most important aspects in all DPD simulations. γ_*ij*_ and σ_*ij*_ are the amplitudes of the dissipative and random forces, respectively. ξ_*ij*_ is a random number with a zero average and unit variance. ω^C^(*r*), ω^D^(*r*), and ω^R^(*r*) are the distance-dependent weight functions. According to the fluctuation–dissipation theorem, ω^D^(*r*) and ω^R^(*r*) are described as follows:

7 $$\omega^{C}\left(r\right)=\{\begin{array}{ll} \left(1-r\right) & \left(r<r_{c}\right)\\ 0 & \left(r\geq r_{c}\right) \end{array}$$

8 $$\omega^{D}\left(r\right)={\left[\omega^{R}\left(r\right)\right]}^{2}=\{\begin{array}{ll} {\left(1-r\right)}^{2} & \left(r<r_{c}\right)\\ 0 & \left(r\geq r_{c}\right) \end{array}$$

9 $$\sigma_{\mathrm{ij}}^{2}=2\gamma_{\mathrm{ij}}k_{B}T$$

where *k*_B_ is the Boltzmann constant, the dissipative parameter (γ_*ij*_) is taken as 4.5, and the random parameter (σ_*ij*_) can be determined by temperature and γ_*ij*_.^47,48^ The subsequent calculations are aimed at obtaining the key parameters *a*_*ij*_ for the conservative force *F*_*ij*_^C^. In a sense, this connects the conservative force in DPD with the forces used for MD calculations. The relationship between *a*_*ij*_ and the Flory–Huggins parameter (*χ*_*ij*_) is as follows:^45,48^

10 $$a_{\mathrm{ij}}=166.67+3.27\chi_{\mathrm{ij}}\left(\rho =3\right)$$

Here, the Flory–Huggins parameter (*χ*_*ij*_) can be calculated from the solubility parameters by the equation:^49^

11 $$\chi_{\mathrm{ij}}=\frac{V_{\mathrm{bead}}}{k_{B}T}{\left(\delta_{i}-\delta_{j}\right)}^{2}$$

where *V*_bead_ is the average volume of the two beads and δ_*i*_ and δ_*j*_ are the solubility parameters of beads *i* and *j*, respectively. The values of solubility parameters of beads can be obtained by molecular dynamics simulations using the COMPASS II force field, and the charge balance method used is assigned by the COMPASS II force field.^50,51^ The Berendsen algorithm with an attenuation constant of 0.1 ps is used to control the temperature. First, a cell consisting of 50 identical beads is constructed. The water cell density is set to 1.0 g/cm^3^, and Type 1 (T1) and Type 2 (T2) cell densities are set to 0.9 g/cm^3^. Second, the energy minimization with an energy tolerance of 2.0 × 10^–5^ kcal/mol and a force tolerance of 1.0 × 10^–3^ kcal/(mol·Å) is performed. Third, a total time of 400 ps under NVT simulations at 275 K is conducted with the integration step of 1.0 fs. Finally, the cohesive energy density and solubility parameters between the beads can be obtained, as shown in Tables 1 and 2.

**Table 1:** Parameters and Composition of Different Beads at 275 K

bead	composition	volume V (Å^3^)	solubility parameter δ (MPa^0.5^)
T1	CH_4_ + 5H_2_O	195.78	42.709
T2	CH_4_ + 6H_2_O	229	43.184
W	6H_2_O	180	48.445

**Table 2:** Interaction Parameters *a*_*ij*_ between Beads at 275 K

bead	T1	T2	W
T1	166.67	166.71	171.97
T2		166.67	171.54
W			166.67

### Simulation Models

The construction of coarse-grained models of molecules is an essential step in DPD simulations. The model in this study is based on the structural characteristics of structure I methane hydrate. Structure I methane hydrate consists of 46 water molecules and 8 methane molecules, and it is composed of two cage types: 5^12^ and 5^12^6^2^. The structure I hydrate structure is coarse-grained into two distinct bead types: one type represents the 5^12^ cage composed of one methane molecule and five water molecules, and the other type represents the 5^12^6^2^ cage composed of one methane molecule and six water molecules. The central points of the T1 and T2 beads correspond to the locations of methane molecules in 5^12^ and 5^12^6^2^ cages, as shown in Figure 1. The prime positions of the oxygen atoms of water molecules were taken from the X-ray diffraction analysis data and the prime positions of the guest molecules are placed in the central area,^12,52^ Moreover, some detailed parameters of the water and methane composing coarse-grained beads are presented as shown in Table S1. The coordinates of the single-crystalline cell used to build hydrate particles are shown in Table S2. Based on this idea, first, the preparation of hydrate particles can be achieved by replicating the initial single crystals in the *x*-, *y*-, and *z*-directions. Then, specific shapes of hydrate particles can be customized by cutting. Finally, water beads are added to the simulation boxes to meet the number density requirements of the DPD method. Furthermore, to ensure comparable volumes for both cage types, six water molecules are grouped together as a single water bead (W) during coarse-graining. In order to maintain the relative positions of different beads within hydrate particles, all particles are set as rigid bodies. This study established four groups, as shown in Table 3. **Sections A1–A4** represent simulated groups related to particle size with particle radii of 10, 8, 6, and 4 nm, as shown in Figure 2. **Sections B1** and **B2** investigate the impact of particle size ratios on particle agglomeration with particle size ratios of 2:1 and 5:1. For example, in **Section B1**, the particle radii are 10 and 5 nm, respectively, as shown in Figure 2b. **Sections C1** and **C2** aim to explore the effect of the initial positions of elliptical particles on particle agglomerations. The volume of elliptical particles is established to be equivalent to that of spherical particles with a radius of 10 nm to examine the effect of particle shape on the agglomeration process. **Sections D1** and **D2** investigate the effect of particle number on particle agglomeration, as shown in Figure 2e,f. The distance between adjacent methane hydrate particles is set to 5 nm. Afterward, a series of models is constructed, as shown in Table 3.

**Figure 1:**
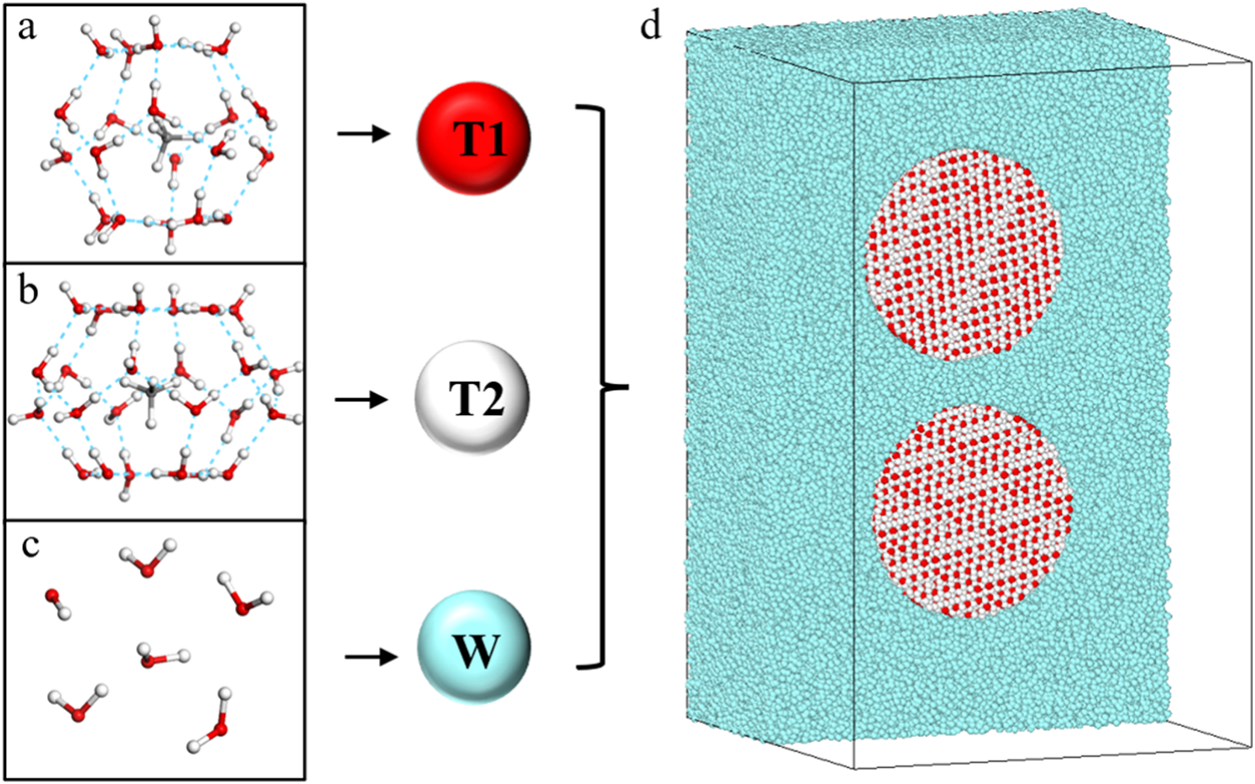
Coarse-grained scheme of different beads to construct hydrate particle systems in aqueous solutions. (a) One 5^12^ cage containing a methane molecule. (b) One 5^12^6^2^ cage containing a methane molecule. (c) Six H_2_O molecules. (d) Profile of the initial structures of two methane hydrate particles with a radius of 10 nm in aqueous solutions (**Section A1**). All water beads, T1 beads, and T2 beads are colored cyan, red, and white, respectively.

**Figure 2:**
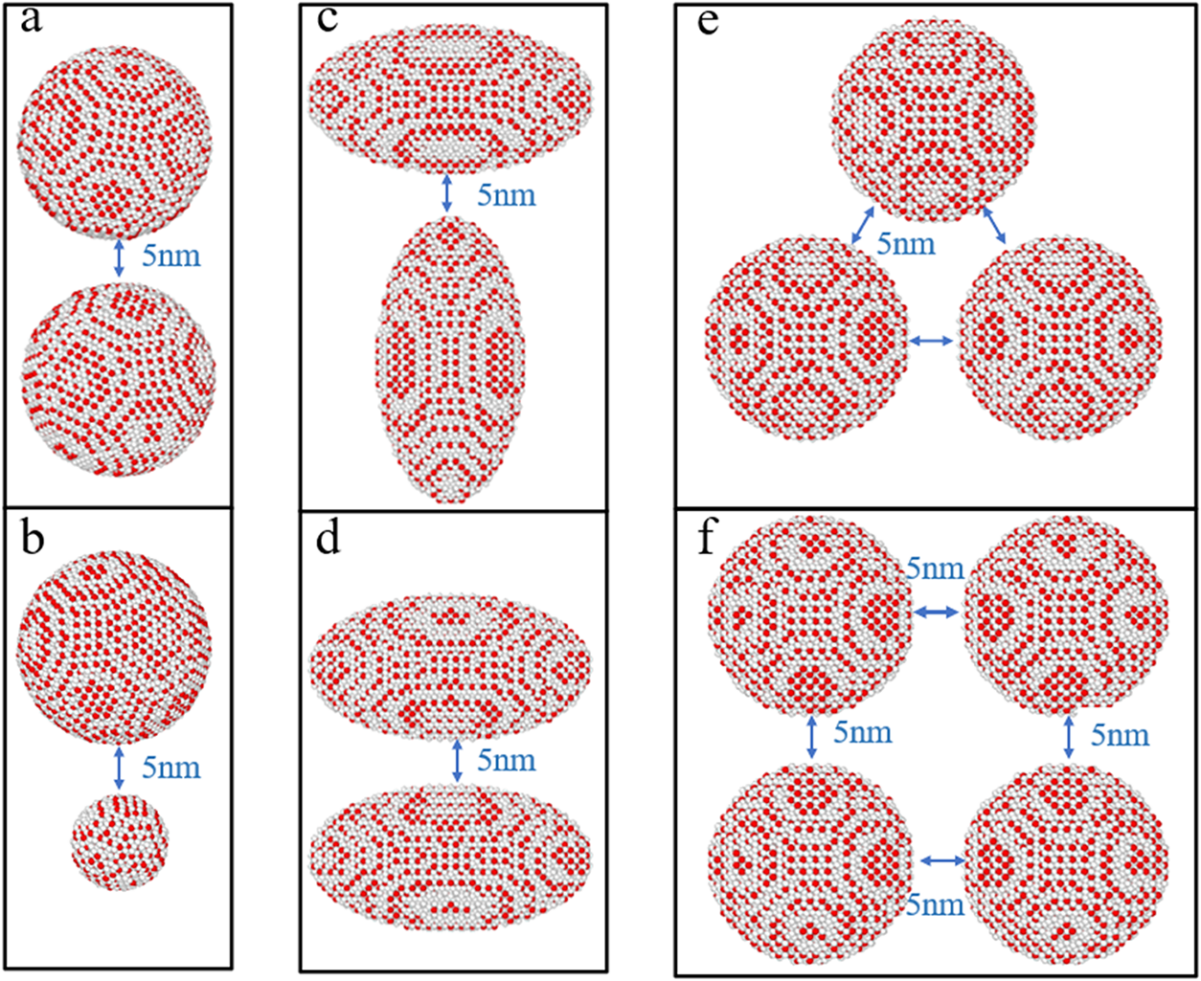
Initial structures in DPD simulations. (a) Two spherical methane hydrate particles with a radius of 10 nm, corresponding to **Section A1.** (b) Two spherical methane hydrate particles with a radius of 10 and 5 nm, respectively, corresponding to **Section B1**. (c) Two ellipsoidal methane hydrate particles with dimensions of 8 nm × 8 nm × 15.6 nm placed vertical to each other, corresponding to **Section C2**. (d) Two ellipsoidal methane hydrate particles with dimensions of 8 nm × 8 nm × 15.6 nm placed parallel to each other, corresponding to **Section C1**. (e) Three spherical methane hydrate particles with a radius of 10 nm, corresponding to **Section D1**. (f) Four spherical methane hydrate particles with a radius of 10 nm, corresponding to **Section D2**. The water beads are not shown for the sake of clarity. All T1 and T2 beads are colored red and white, respectively.

**Table 3:** Parameters of Constructed Models in Our DPD Simulations

group	particle number	particle size ratio	particle size (nm)	particle shape	box size (*R*_c_)
**Section A1**	2	1:1	10 (radius)	spheroid	50 × 50 × 80
**Section A2**	2	1:1	8 (radius)	spheroid	50 × 50 × 80
**Section A3**	2	1:1	6 (radius)	spheroid	50 × 50 × 80
**Section A4**	2	1:1	4 (radius)	spheroid	50 × 50 × 80
**Section B1**	2	2:1	10:5 (radius)	spheroid	50 × 50 × 80
**Section B2**	2	5:1	10:2 (radius)	spheroid	50 × 50 × 80
**Section C1**	2		8 × 8 × 15.6	ellipsoid	80 × 80 × 40
**Section C2**	2		8 × 8 × 15.6	ellipsoid	80 × 80 × 40
**Section D1**	3	1:1:1	10 (radius)	spheroid	50 × 70 × 100
**Section D2**	4	1:1:1:1	10 (radius)	spheroid	50 × 70 × 100

### Simulation Details

All DPD simulations are performed using the massively parallelized large-scale atomic/molecular massively parallel simulator (LAMMPS) package.^53^ The number density ρ of all simulations is chosen to be 3, and all three directions of the box have periodic boundary conditions. The cutoff *r*_c_, bead mass m, and thermal energy *k*_B_ *T* (= 1) serve as *lj* units for length, mass, and energy, respectively, in the input script. Based on the water bead volume, *r*_c_ is set to $\sqrt[{3}]{3V_{\mathrm{bead}}}=\sqrt[{3}]{3\times 180}$ = 8.143 Å at *ρ* = 3. The DPD time scale is $\tau =r_{C}\sqrt{\frac{m}{k_{B}T}}=8.143\times 10^{-10}\times \sqrt{\frac{108\times 10^{-3}}{1.38\times 10^{-23}\times 275\times 6.02\times 10^{23}}}=5.599\;\mathrm{ps}$ . All quantities used in LAMMPS scripts are in *lj* units, as shown in Table 4. The *lj* units of some basic quantities are converted in Table 4.

**Table 4:** *lj* Unit Conversion Table

basic parameter	dimensionless value	real value
*R*_C_	1.0	8.143 Å
*m*	1.0	108 amu
*k*_B_	1.0	1.380649 × 10^–23^ J/K
*T*	1.0	275 K
ϵ	1.0	0.546 kcal/mol
τ	1.0	5.599 ps
Δ*t*	0.05	0.279 ps

All simulations are performed in the NVT ensemble at a given simulated temperature of *T* = 1 (*T*_real_ = 275 K). In order to observe the agglomeration phenomenon faster, we set the time step to Δ*t* = 0.05τ. The simulation step is set to be 2 × 10^7^.

## Results

### Agglomerations of Hydrate Particles with Various Particle Sizes

Figure 3 illustrates the agglomerations and structural evolutions of two methane hydrate particles with a radius of 6 nm in aqueous solutions at different simulation times. Three agglomeration stages can be determined from these evolutionary structures. In the first stage, the hydrate particles begin to move and rotate irregularly, and the distance between hydrate particles fluctuates within a range at this stage, as shown in Figure 3a–c. Moreover, both translational and rotational motion dominate hydrate particle behaviors in this stage. In the second stage, the distance between hydrate particles decreases rapidly once methane hydrate particles aggregate together, as shown in Figure 3d. In the last stage, the distance between hydrate particles basically does not change after the occurrence of hydrate particle agglomerations. The contact point of particle agglomeration does not change. Moreover, methane hydrate particles move together in translation and rotation in this stage. The particles coalesce into a unified entity in aqueous solutions, exhibiting reduced motion amplitude compared to those at the first stage. Notably, the hydrate particles remain conjoined throughout the following simulation duration. However, it is noted that only the hydrate particles with a radius of 6 nm in aqueous solutions exhibit a direct contact agglomeration behavior in **Sections A1–A4**, as shown in Figure 3, and Supporting Information Figures S1–S6 and Movies S1, S2, S3 and S4. Therefore, it can be concluded that different agglomerations of methane hydrate particles with different particle sizes can occur based on their evolutionary structures. The Supporting Information Movies S1, S2, S3 and S4 reveal a preagglomeration stage characterized by the translations and rotations of hydrate particles.

**Figure 3:**
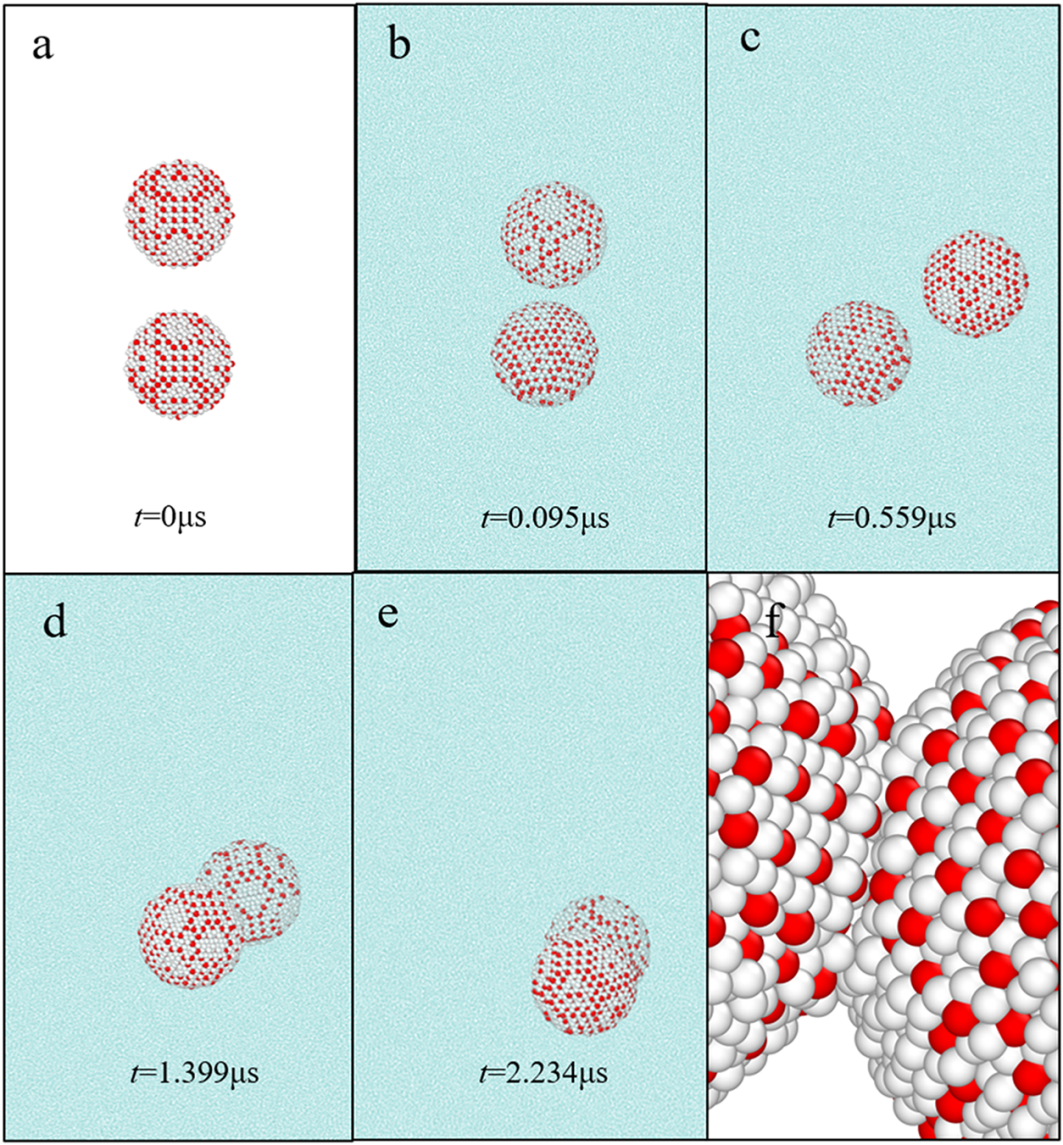
Structural evolutions of two methane hydrate particles with a radius of 6 nm in aqueous solutions. (a–e) Structural evolutions of **Section A3** at different simulation times. (f) Local structure in **Section A3** at 2.234 μs. The water beads in parts (a) and (f) are not shown for clarity. All water beads, T1 beads, and T2 beads are colored cyan, red, and white, respectively.

To further explore the movement of hydrate particles during the agglomeration processes, the distance between the centers of two hydrate particles is quantitatively analyzed. The relationship between the distance among hydrate particles and simulation time during the agglomeration processes of **Section A3** is depicted in Figure 4a. As shown in Figure 4, the particle agglomeration process can be roughly divided into three stages. The initial stage of evolution is characterized by a gradual evolution feature at a relative distance between the hydrate particles. Due to interactions of conservative and dissipative forces, the evolution of hydrate particles exhibits oscillatory motions, resulting in a relatively stable range of distance changes. Moreover, the distance between the hydrate particle centroids of **Section A3** is kept within the range of 13–19 nm. In the second stage, the distance between hydrate centroids decreases rapidly after those hydrate particles randomly reach a critical position. In the last stage, methane hydrate particles could contact each other completely. Interestingly, the contact regions between those hydrate particles remain relatively unchanged once hydrate particles aggregate together, indicating the existence of a binding force between hydrate particles. Based on the distance between hydrate particle centroids at this stage, it is difficult for the hydrate particles in aqueous solutions to overcome the binding force to separate them without external forces. In terms of their motion behaviors, the assembled hydrate particles exhibit strong structural stability. Figure 4b shows the distribution of DPD beads along the *Z*-direction at different simulation times. Two distinct valleys at around 0.089 μs can be attributed to the distance between the two hydrate particles before they are united. It is worth noting that the density of the methane hydrate particles is slightly lower than that of water. With increasing simulation time, two hydrate particles merge to form a unified mass, resulting in the appearance of a single valley in the number of bead along the *Z*-direction at about 2.228 μs. Agglomerations of hydrate particles in **Sections A1**, **A2**, and **A4** do not occur. Only the first stage can be observed on the relative distance between methane hydrate particle centroids, as shown in Supporting Information Figures S2, S4, and S6. In addition, based on the relative distance of the centroids of methane hydrate particles in **Sections** **A1**, **A2**, and **A4**, it is found that the relative distances in **Sections A1** and **A2** oscillate occasionally, then gradually decrease, and finally remain within a very small range, analogous to those of **Section A3.** However, the relative distances in **Section** **A4** show a large oscillation amplitude and a high oscillation frequency, demonstrating strong randomness.

**Figure 4:**
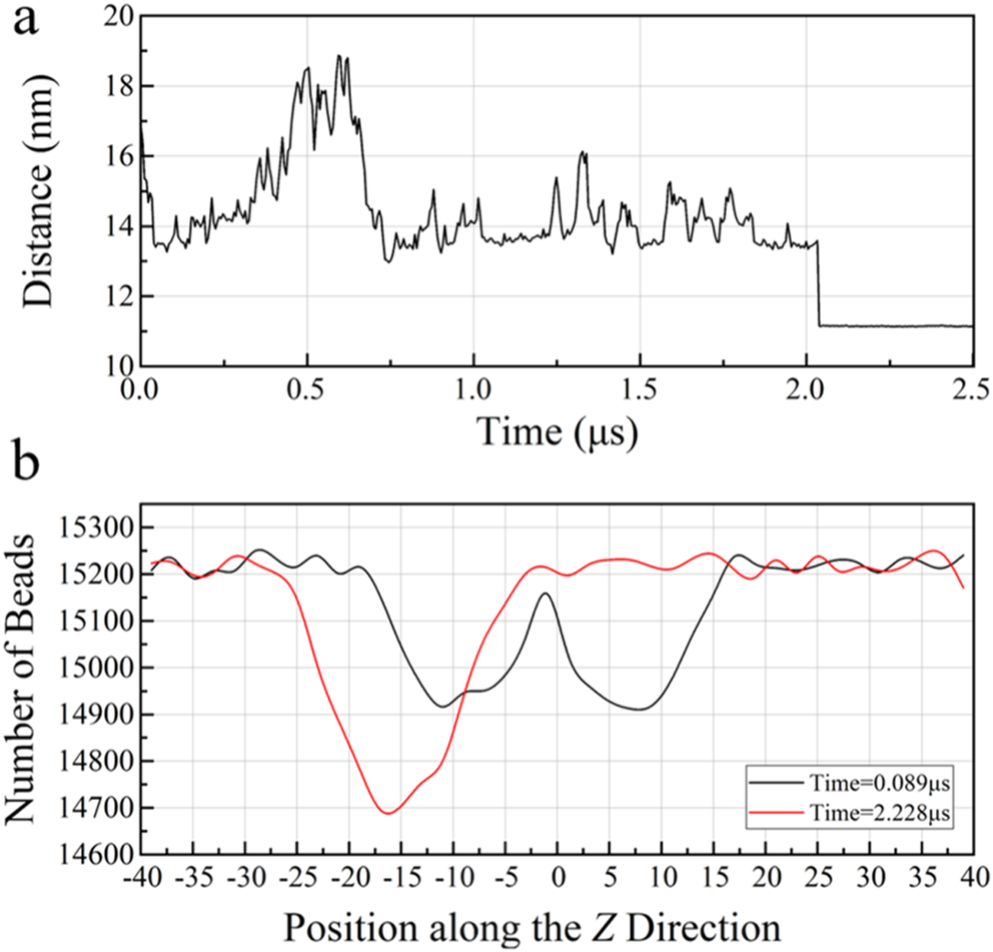
Evolutions of parameters of two methane hydrate particles with a radius of 6 nm in aqueous solutions. (a) Relative distance between methane hydrate particle centroids as a function of simulation time in **Section A3**. (b) Number of beads along the *Z*-direction at different simulation times in **Section A3**.

### Particle Size Ratio-Controlled Agglomerations

To understand the particle size ratio-controlled agglomerations of hydrate particles, the structural evolutions of methane hydrate particles with particle size ratios of 2:1 and 5:1 in aqueous solutions are captured at different simulation times in **Sections B1** and **B2**, respectively. As shown in Figure 5, particle agglomerations of methane hydrates are found in **Sections B1** and **B2.** Such particle agglomerations are similar to those observed in methane hydrate particle systems with particle size ratios of 1:1 in aqueous solutions in **Section A3.** Moreover, it can be found that particle agglomerations occur within a shorter time in **Sections B1** and **B2** than those in **Section A3**, as shown in Figures 6a and S7. Three agglomeration stages can also be found in these evolutionary structures. However, it can be seen that the oscillation time at the first stage in **Section B2** with a particle size ratio of 5:1 is longer than that in **Section B1** with a particle size ratio of 2:1. This is similar to the shock imbalance in Brownian motion,^54^ in which the different particle sizes and forces from the water drop can lead to different imbalances. In the second stage, the relative distances of the centroids of methane hydrate particles in **Sections B1** and **B2** were also different. **Section B2**, with a particle size ratio of 5:1, has a considerable recovery distance. After particle agglomeration, the distances between hydrate particle centroids in **Sections B1** and **B2** do not change. The third stage of **Sections B1** and **B2** is analogous to that in **Section A3**. In addition, it can be observed that **Section B1** has two valleys at 0.084 μs before particle agglomeration, representing the existence of a relative distance between two hydrate particles with different particle sizes, as shown in Figure 6b. However, **Section B2** has only one valley, which is caused by the small particle size of one hydrate particle in **Section B2**. Based on the basic theory and model construction of this simulation, the particle agglomeration process in this simulation may be different from the sintering mechanism,^55^ and the conversion of hydrate cannot occur after the agglomeration is limited by the DPD model in this work. Based on the relative distance of the centroids of methane hydrate particles in **Sections B1** and **B2**, it is found that the relative distance in **Section B1** has a very small oscillation amplitude during the oscillation stage. However, the relative distance in **Section B2** exhibits a large oscillation amplitude during the oscillation stage.

**Figure 5:**
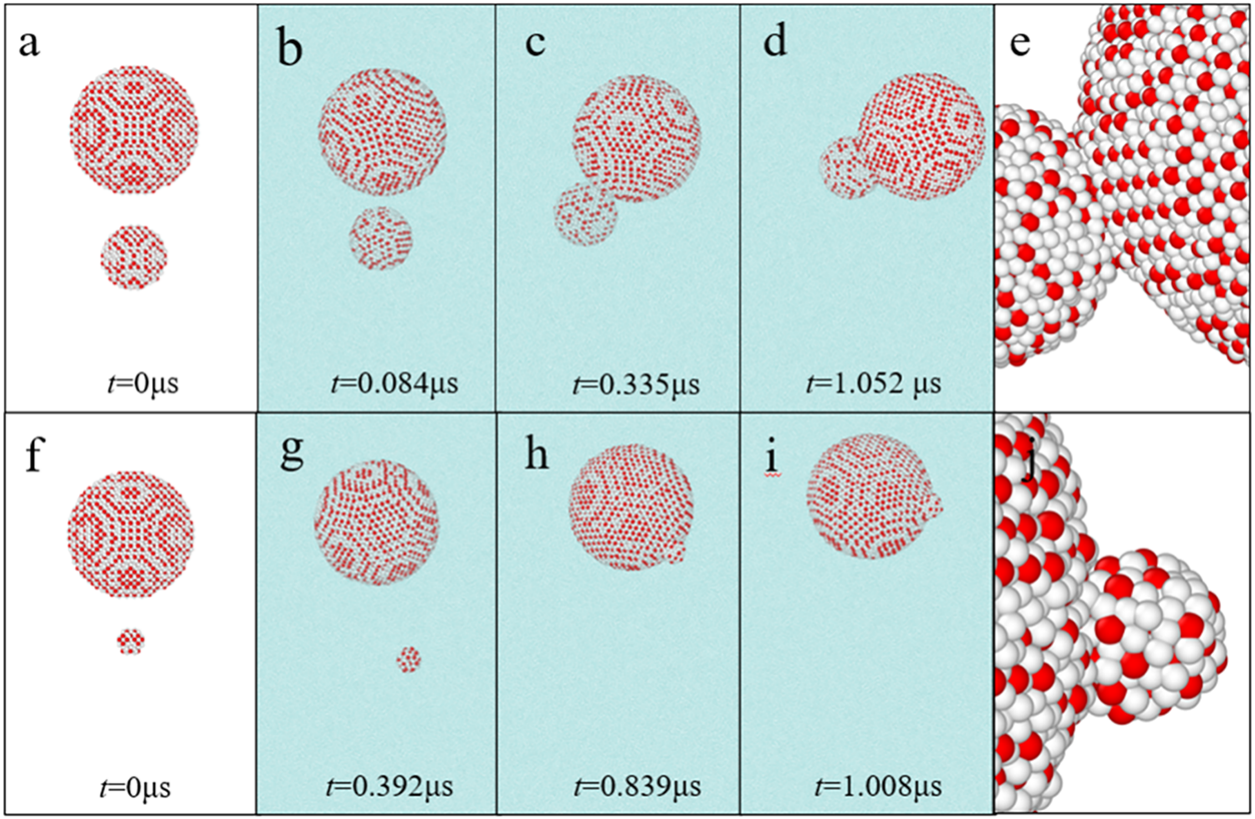
Structural evolutions of two methane hydrate particles with different particle size ratios in aqueous solutions. (a–d) Structural evolutions of **Section B1** in aqueous solutions at different simulation times. (e) Local structure in **Section B1** at 1.052 μs. (f–i) Structure evolutions of **Section B2** in aqueous solutions at different simulation times. (j) Local structure in **Section B2** at 1.008 μs. The water beads in (a, e, f, j) are not shown for clarity. All water beads, T1 beads, and T2 beads are colored cyan, red, and white, respectively.

**Figure 6:**
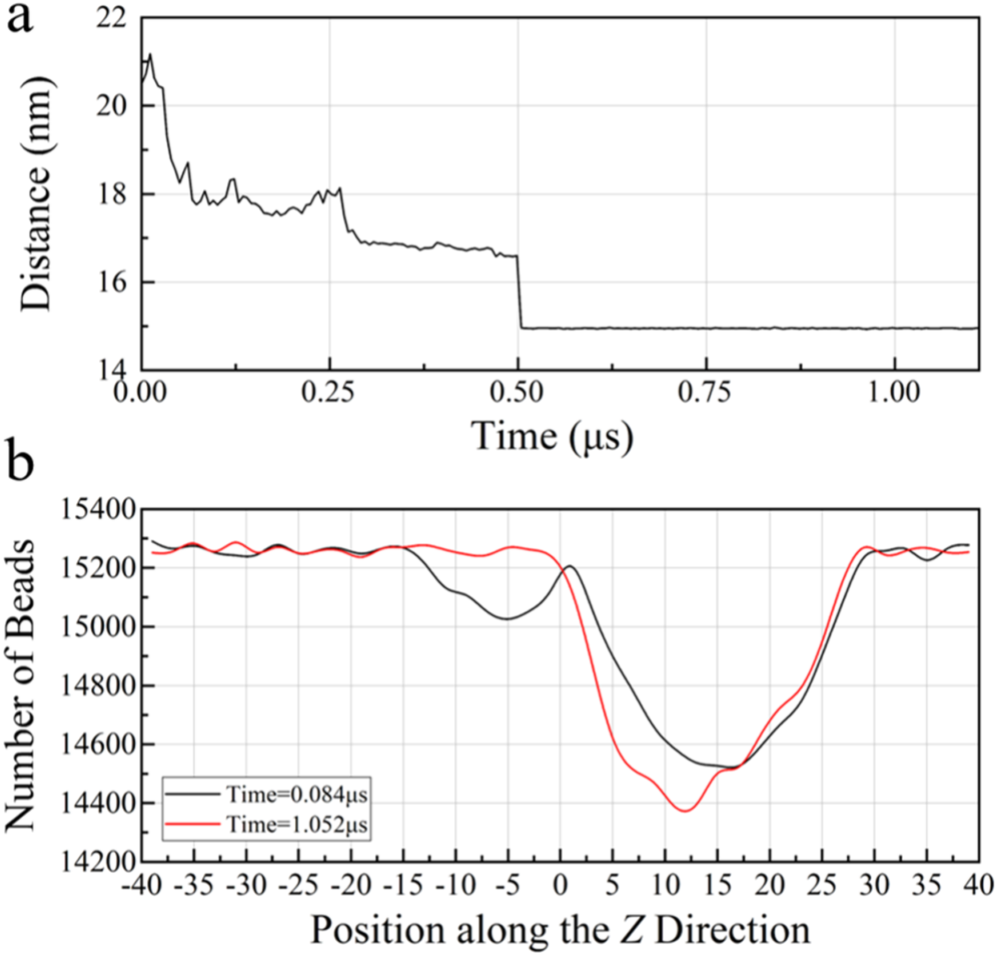
Evolutions of the parameter of two methane hydrate particles with a radius of 10 and 5 nm, respectively, in aqueous solutions. (a) Relative distance between methane hydrate particle centroids as a function of simulation time in **Section B1**. (b) Number of beads along the *Z*-direction at different simulation times in **Section B1**.

### Evolutionary Differences in Ellipsoidal and Spheroidal Hydrate Particles

Gas hydrate particles typically have irregular shapes, and it is important to study the effect of their shapes on agglomerations of methane hydrate particles by DPD simulations. Figures 7 and S8 show the structural evolutions of two ellipsoidal methane hydrate particles in aqueous solutions in **Sections C1** and **C2**. Although the ellipsoidal hydrate particles are placed under specific conditions at different angles, there are similarities in their movement processes, as shown in Figures 7 and S8. Whether methane hydrates are placed parallel or vertical to each other first, the ellipsoidal hydrate particles will gradually move to the offset parallel relative positions, as shown in Figures 7d and S8e. This indicates that the parallel relative positions of two ellipsoidal methane hydrate particles may be the relatively stable configurations that two ellipsoidal hydrate particles can achieve. The relative distance of the centroids of ellipsoidal methane hydrate particles in **Section C1** exhibits oscillatory and declining behaviors as shown in Figure 8a. After about 1.2 μs, two ellipsoidal methane hydrate particles reach a metastable agglomeration state without direct contact, with the distance basically maintained within the range of about 18.5 to 19 nm. This process is similar to the changes in **Sections** **A1** and **A2**, which is a noncontact metastable agglomeration of hydrate particles maintaining their respective motion characteristics. This noncontact metastable agglomeration shows differences in morphology from the direct contact agglomeration of **Sections** **A3, B1**, and **B2**. After the occurrence of contact agglomerations, the contact points of the hydrate particles remain basically unchanged, and there is no change in the relative distance between hydrate particles as they move as a whole. However, as for noncontact metastable agglomerations, the two particles rotate separately, maintaining a certain distance. By comparison, it is found that free movements of ellipsoidal methane hydrate particles can occur in **Section C2** within the simulation time, as shown in Figures S8 and S9. Moreover, based on those results, methane hydrate particles with different particle sizes prefer to aggregate together, while the agglomeration trend of ellipsoidal particles is not obvious. Those also indicate that the hydrate particle shapes can affect the hydrate agglomeration processes.

**Figure 7:**
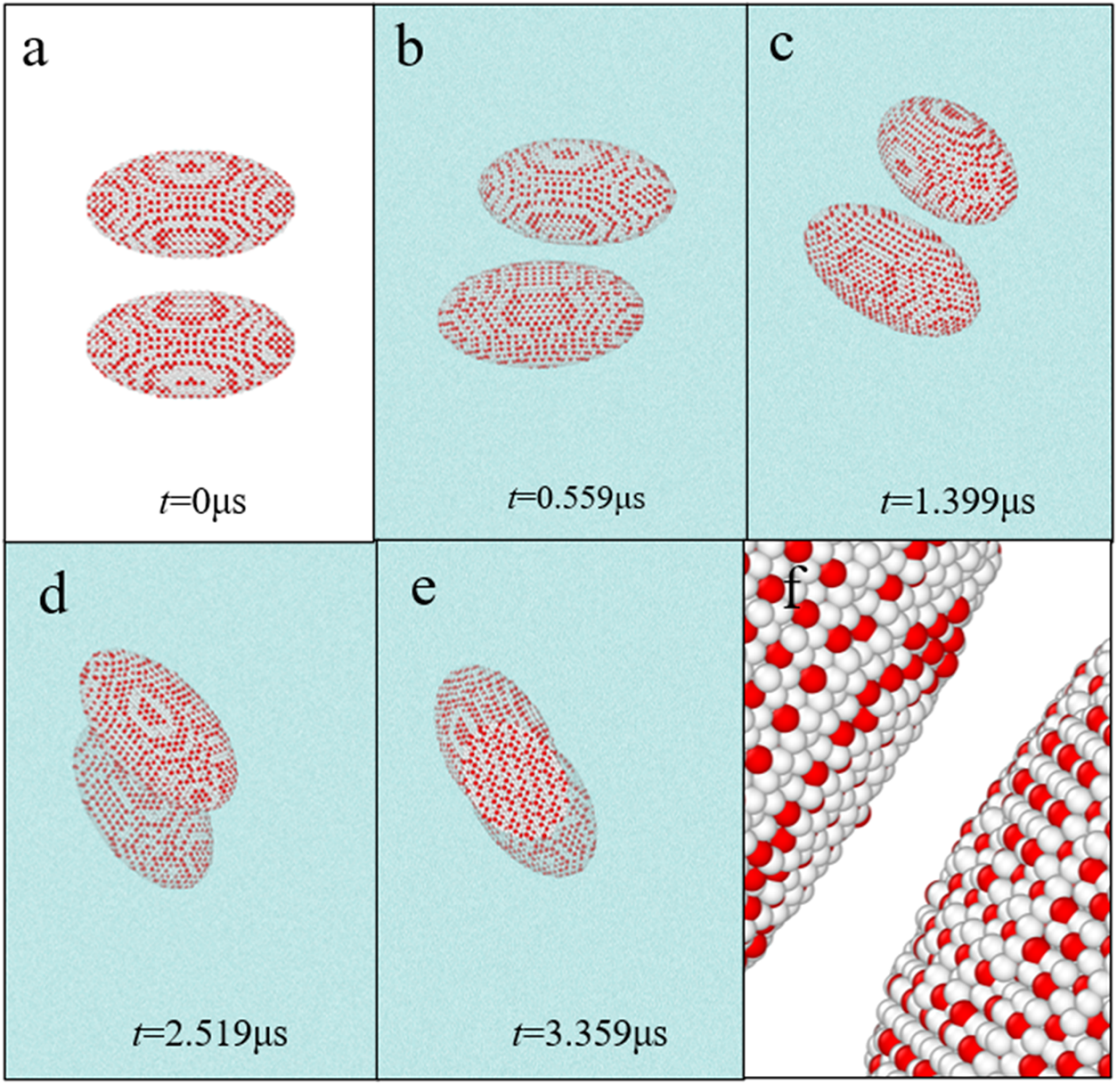
Structural evolutions of two ellipsoidal methane hydrate particles with dimensions of 8 nm × 8 nm × 15.6 nm placed parallel to each other in aqueous solutions. (a–e) Structural evolutions of **Section C1** in aqueous solutions at different simulation times. (f) Local structure in **Section C1** at 3.359 μs. The water beads in (a, f) are not shown for clarity. All water beads, T1 beads, and T2 beads are colored cyan, red, and white, respectively.

**Figure 8:**
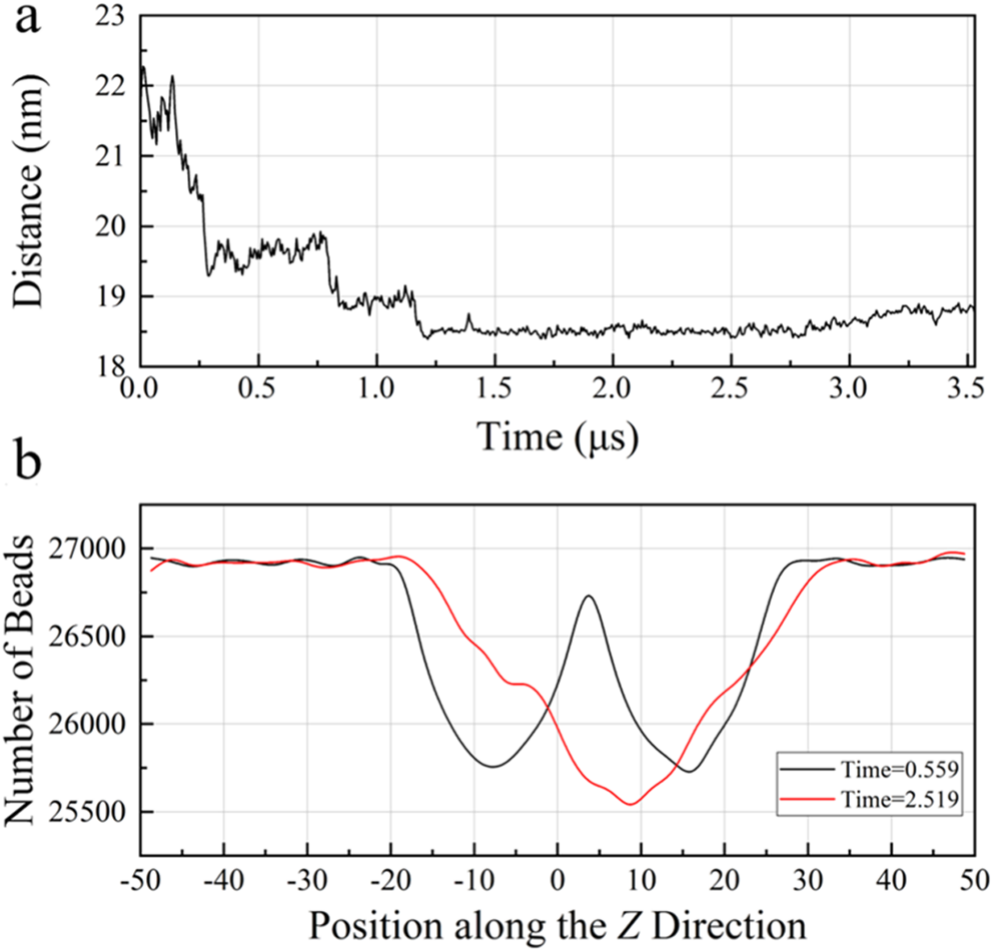
Evolutions of parameters of two ellipsoidal methane hydrate particles with dimensions of 8 nm × 8 nm × 15.6 nm placed parallel to each other in aqueous solutions. (a) Relative distance between methane hydrate particle centroids as a function of simulation time in **Section C1**. (b) Number of beads along the *Z*-direction at different simulation times in **Section C1**.

### Agglomeration Patterns in Multiparticle Systems

As shown in Figures 9 and S10, the noncontact metastable agglomerations mentioned earlier also occur in **Sections** **D1** and **D2**, and their relative distances of the centroids of methane hydrate particles show very small fluctuations after the occurrence of noncontact metastable agglomerations. At approximately 2 μs, methane hydrate particles in **Section D2** show noncontact metastable agglomerations. As shown in Figure 9a–c, the four hydrate particles initially approach each other, then they form the noncontact metastable agglomeration structure shown in Figure 9d. Indeed, there is no structural overlapping in **Sections A1**, **A2, C1, D1**, and **D2**. Such an illusion of structural overlapping is caused by observing visual direction, demonstrated by the relative distance between the methane hydrate particle centroid as a function of simulation time in **Sections A1**, **A2, C1, D1**, and **D2**. Within a period of 1.12 μs, as shown in Figure 9d,e, the change of structural evolutions is very small; those hydrate particles move slightly as a whole. In addition, for two-particle, three-particle, and four-particle systems, there are one, three, and six sets of relationships between distance and simulation time, respectively. When all distances in all sets reach stable states, the state of hydrate particle agglomerations can be defined. As shown in Figure 10a, the distances in six sets are basically stable at around 22 nm at 2 μs. The number of beads along the *Y*-direction in Figure 10b also supports this point as the simulation time increases from 0.279 to 2.239 μs. Similar behaviors are also found in **Section D1**, as shown in Figure S11. Does the increase in the number of hydrate particles also increase the probability of agglomeration in an imperceptible way? An increase in the number of hydrate particles in boxes of the same size can increase the probability of particle collisions. Moreover, **Sections** **A3, B1**, and **B2** prove that hydrate particles will be closely connected together after particle collisions. Therefore, the number of hydrate particles to a certain extent has an impact on agglomerations, resulting in the large probability of the occurrence of hydrate particle agglomerations.

**Figure 9:**
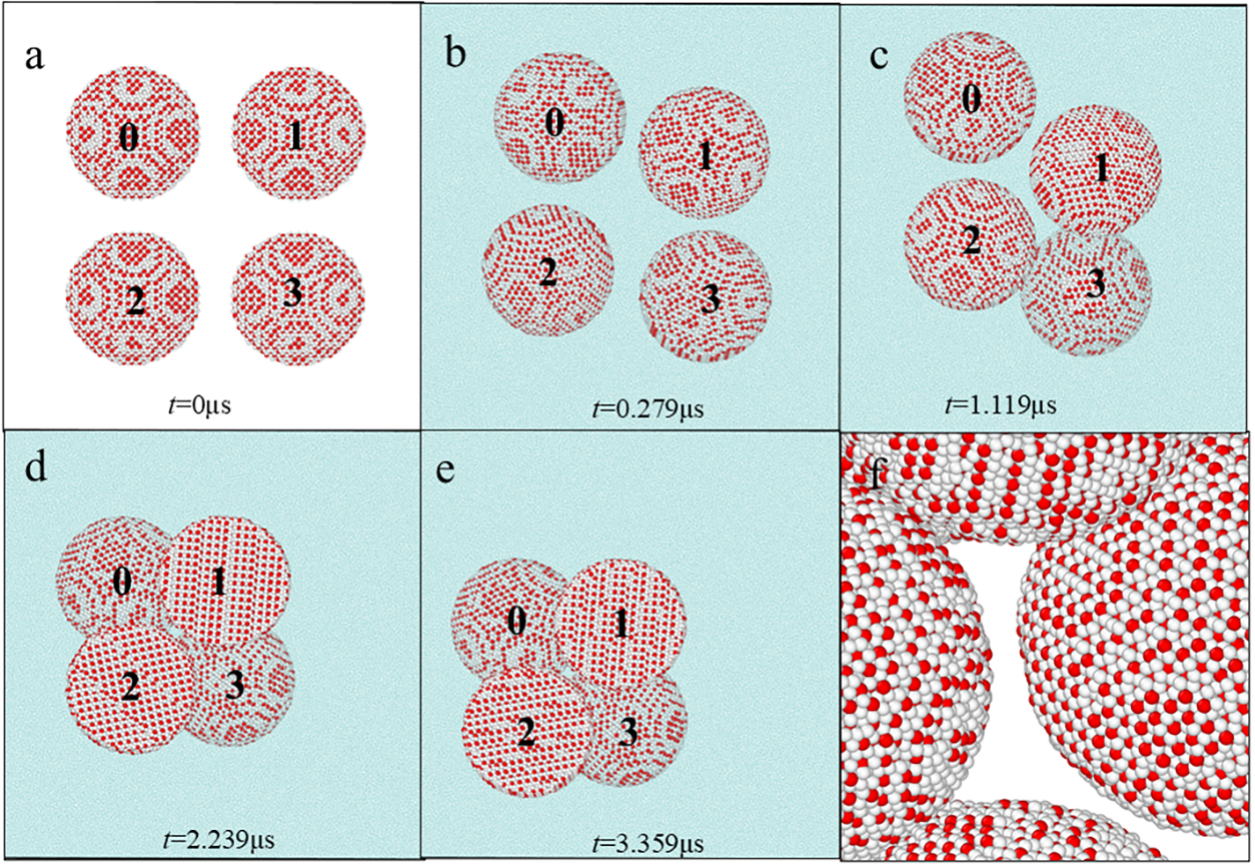
Structural evolutions of four spherical methane hydrate particles with a radius of 10 nm in aqueous solutions. (a–e) Structural evolutions of **Section D2** in aqueous solutions at different simulation times. (f) Local structure in **Section D2** at 3.359 μs. The water beads in (a, f) are not shown for clarity. All water beads, T1 beads, and T2 beads are colored cyan, red, and white, respectively.

**Figure 10:**
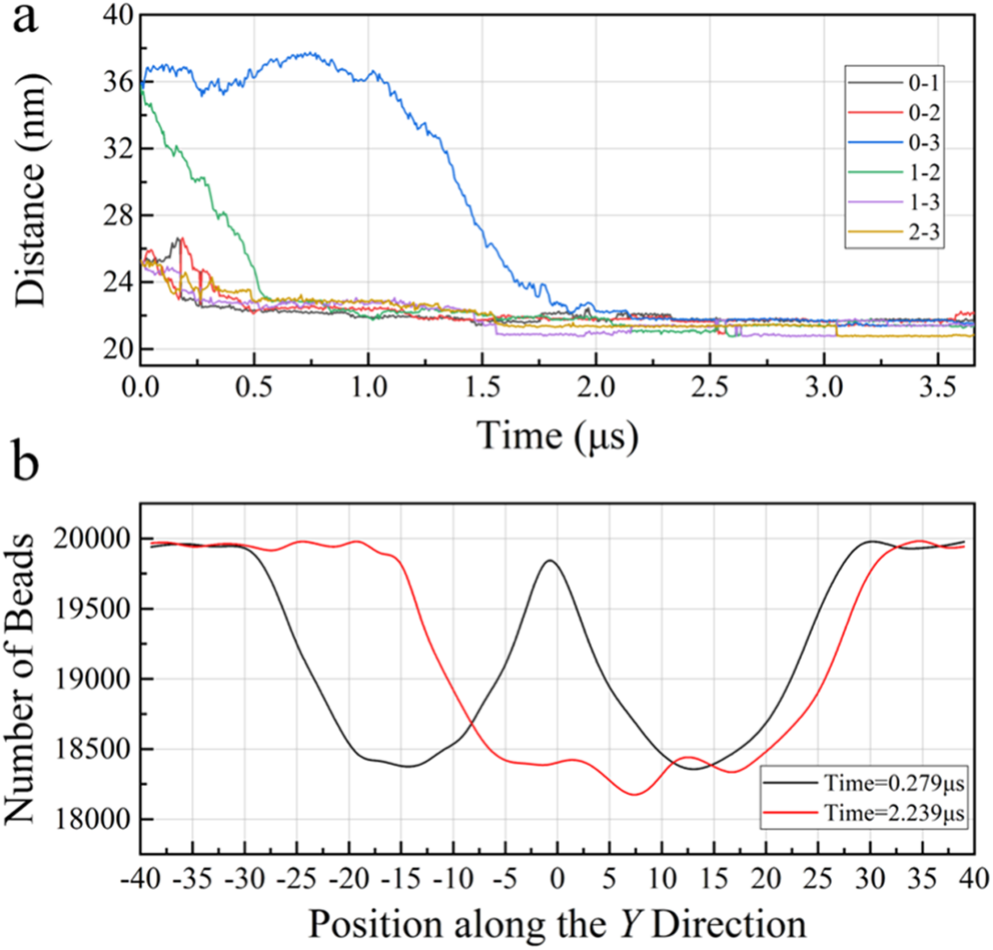
Evolutions of parameters of four spherical methane hydrate particles with a radius of 10 nm in aqueous solutions. (a) Relative distance between methane hydrate particle centroids as a function of simulation time in **Section D2**. (b) Number of beads along the *Y*-direction at different simulation times in **Section D2**.

## Discussions

To the best of our knowledge, a fundamental understanding of gas hydrate particle agglomeration is essential for flow assurance and safety issues in oil and gas pipelines. In this work, different particle agglomeration patterns, e.g., stable agglomeration state with direct contact (SASDC), metastable agglomeration state without direct contact (MASDC), and free state, have been discovered. Upon SASDC, it can be attributed to a larger driving force than a repulsive force, as shown in Figure 11. As described by the DPD method, the conservative, dissipative, and random forces synergistically dominate the motions of beads. Moreover, the conservative force is a soft repulsive interaction force. This suggests that gas hydrate particle agglomeration results from the collective behavior of all beads in the DPD system. Furthermore, this SASDC agglomeration is analogous to a bonding process, characterized by the solid bridge force playing a dominant role in particle agglomeration. In the SASDC agglomeration, hydrate agglomerates exhibit no separation once formed. For example, no further significant structural changes occurred in the agglomerates of **Sections** **A3, B1**, and **B2**. As for the MASDC, it results from a driving force equal to a repulsive force. A liquid bridge may play a crucial role in the MASDC agglomeration. Recent studies have proposed that a premelted quasi-liquid layer exists on hydrate surfaces even below the bulk melting point.^56,57^ The capillary attraction induced by quasi-liquid layer can generate significant adhesion force between hydrate particles or between hydrate and solid surfaces.^58^ This attraction induced by the quasi-liquid layer likely constitutes a key component of the driving force in our work. Furthermore, the hydrate particle aggregations in MASDC, which is attributed to a liquid bridge, align with the content that the quasi-liquid layer facilitates particle interfacial consolidation. The force from the liquid bridge is related to the distance between particles^59^ and the volume of the liquid bridge.^60^ Moreover, the shape of the liquid bridge is related to the shape of the particles, suggesting the distinctive force field around hydrate particles with different shapes. Essentially, the interaction parameters (*a*_*ij*_) between T1/T2 and W beads are slightly higher than those between water beads, resulting from each T1/T2 bead being represented by a water cage containing a methane molecule. Moreover, T1/T2 beads can be regarded as hydrophobic beads due to their differential interaction parameters compared to water beads. Interestingly, the hydrophobic tail of phospholipid molecules can spontaneously gather together in water.^61^ Such interactions between T1/T2 and W beads play an important role in the agglomerations of methane hydrate particles in aqueous solutions. With respect to the free state, it represents the free movement of methane hydrate particles by translation and rotation without the agglomeration phenomenon, as shown in Figures S5, S6, S8, and S9. For example, **Section A4** demonstrates the randomness of hydrate particle movement by translation and rotation. The small hydrate particles in **Section A4** can be greatly affected by the interactions with water beads, leading to a smaller driving force than the repulsive force, as shown in Figure 11. Three particle agglomeration patterns, including SASDC, MASDC, and free state, are found in **Sections A1–A4**, depending on the particle size of the hydrate particles. Such particle size-controlled particle agglomeration patterns are analogous to the phenomenon characterized by the Stokes–Einstein equation.^62^ For example, small particles often show large diffusion coefficients at the same external interactions. Small particles frequently approach or move away from large particles due to their fast diffusion. Although large particles move slowly, their large volume increases the possibility of colliding with small particles. Moreover, the effect of collisions in systems with different particle sizes is not significant,^63^ suggesting different agglomerations because of different particle sizes. This asymmetric motion pattern further governs the probability of the agglomeration phenomenon of hydrate particles. This further explains different agglomerations in **Sections A1–A4**. Throughout the entire simulation process, hydrate particles first move randomly in the system by translation and rotation, consistent with the previous molecular simulations.^39^ As revealed by previous experimental and simulation data,^39,64–68^ various agglomerations of hydrate particles are also found. For example, Wang et al.^64^ studied the agglomeration of micron-sized hydrate particles in pure water systems, focusing on the shear agglomeration mechanism in the flow field. Duan et al.^65^ simulated agglomerations of millimeter-scale hydrate particles in a water-dominated system using the CFD-DEM simulation method. Yu et al.^68^ found a small amount of large particle agglomerations and a relatively high concentration of medium-sized agglomeration particles, indicating the diversity of particle agglomerations. Crucially, the works by Nguyen et al.^56–58^ provide a fundamental interface-focused explanation for the origin of interparticle adhesion. In this work, different particle agglomeration patterns can occur due to different driving forces and repulsive forces.

**Figure 11:**
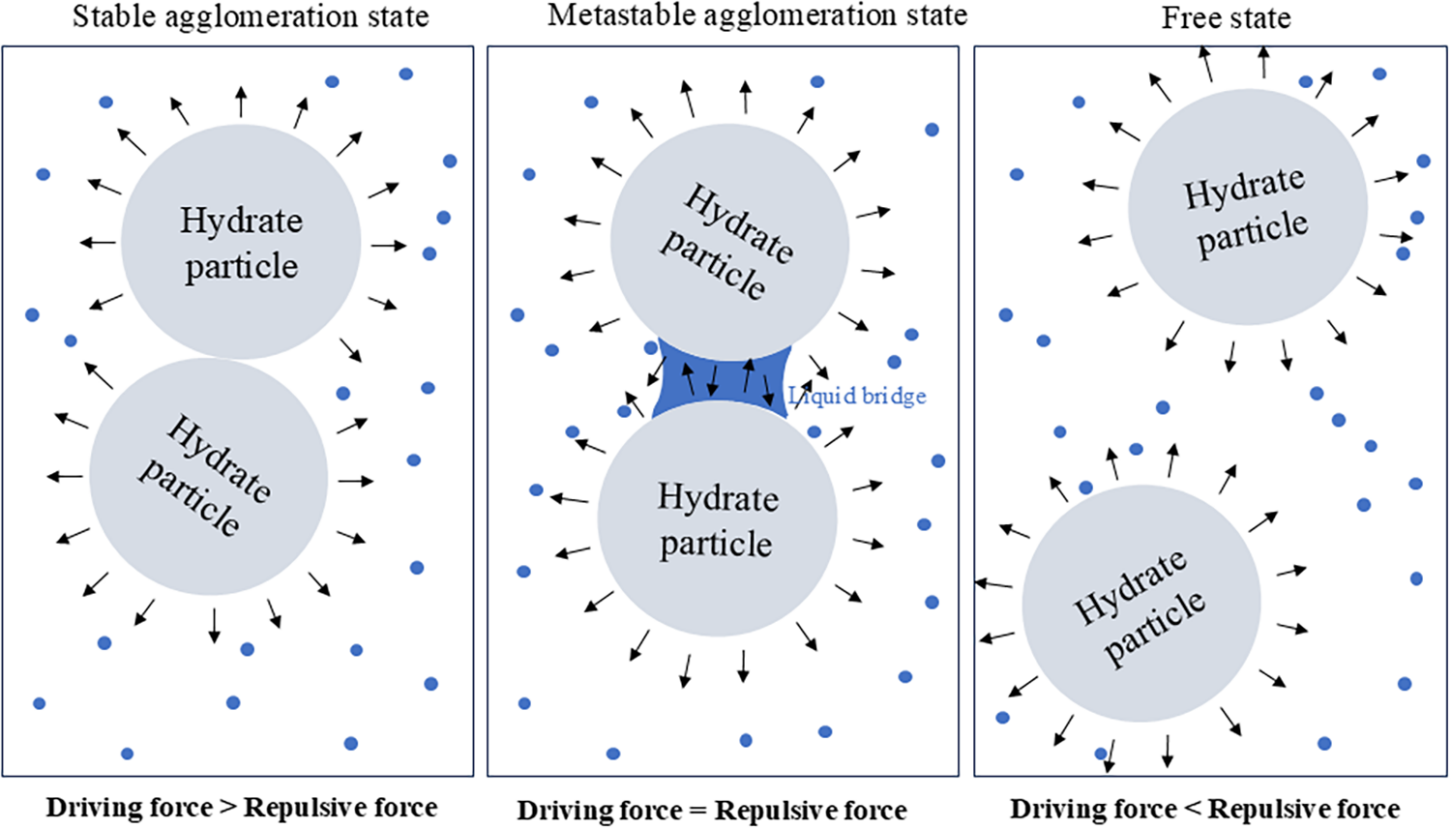
Schematic diagrams of agglomeration mechanisms of methane hydrate particles. The black arrow represents the repulsive force field around the hydrate particles.

To provide important physical insights into the agglomeration of methane hydrate particles, some physical properties, e.g., mean square displacement (MSD), diffusion coefficients, and local structures, are captured as shown in Figures S12 and S13 and Table S4. Upon MSD of water beads, it is increased with increasing simulation time, as shown in Figure S12. Moreover, the difference in MSD among various methane hydrate particle systems in aqueous solutions can be observed, indicating the potential distinctive role of methane hydrate particles on the MSD of water beads. The diffusion coefficients of water beads have also been computed based on the MSD of the water beads, as shown in Table S4. The diffusion coefficient of water beads in each methane hydrate particle system maintains a basic constant value, resulting from the constant temperature at those simulations in this work. Meanwhile, the distance changes between methane hydrate particles can show the diffusion phenomenon of methane hydrate particles. For local structures in the regions between hydrate particles, they are drawn to further understand the agglomeration evolution of hydrate particles. As shown in Figure S13, the water beads are distributed around the connection area. When the hydrate particles gather, they still maintain their original crystal arrangement due to the rigid setting. As shown in Figure S13c,d, the four hydrate particles are very close to each other, exhibiting the nondirect contact manner as a metastable agglomeration state. Moreover, the narrow regions between those hydrate particles are filled with water beads. In our DPD simulations, a fluctuation in the positions of the coarse-grained beads is detected. For example, the positions of the water beads can fluctuate in our simulations. Although the DPD coarse-grained model cannot resolve atomic lattice matching, the coarse-grained method in this study smoothed the dynamic details of hydrogen bonds while retaining the stability of the hydrate structures. Our work provides a new methodological framework for the gas hydrate research community.

## Conclusions

In this study, the dissipative particle dynamics method is used to simulate the agglomeration process of methane hydrate particles under different conditions. The effects of particle size, particle size ratios, particle shape, and particle number on the agglomeration process are studied. The different particle sizes, particle size ratios, and shapes of methane hydrate particles in aqueous solutions can affect their agglomeration evolution. Moreover, the probability of the agglomeration phenomenon can be greatly controlled by them under specific conditions. The number of hydrate particles also affects the agglomerations of hydrate particles. Different particle agglomeration manners are found in those simulation models. The stable agglomeration state with direct contact occurs in **Sections A3, B1**, and **B2**, and the metastable agglomeration state without direct contact is observed in **Sections A1**, **A2, C1, D1**, and **D2**. The free movements of methane hydrate particles by translation and rotation without the agglomeration phenomenon are observed in **Sections A4** and **C2** within the simulation time. These findings highlight the role of hydrate particle size ratios, particle number, and particle shape on the agglomeration process of hydrate particles in aqueous solutions. Our study not only advances our understanding of the agglomeration mechanisms of hydrate particles but also provides valuable insights into flow assurance and safety issues in oil and gas pipelines.

## Supplementary Material























## Data Availability

All data needed to evaluate the conclusions in the paper are present in the paper and/or the Supporting Information. The data that support the findings of this study are available from the cn reasonable request.

## Notes

Structural evolutions of two methane hydrate particles in aqueous solutions; the evolutions of parameter of two methane hydrate particles in aqueous solutions; the structural evolutions of two ellipsoidal methane hydrate particles in aqueous solutions; the evolutions of parameter of two ellipsoidal methane hydrate particles; the structural evolutions of three spherical methane hydrate particles in aqueous solutions; the evolutions of parameter of three spherical methane hydrate particles in aqueous solutions; the mean square displacement of water beads at various simulation times; local structures of methane hydrate particles in aqueous solutions; the detail parameters of the molecular model; the initial coordinate information of T1 and T2 in the single-crystalline cell; summary table of agglomeration results in all sections; summary table of diffusion coefficients of water beads; and visualizations of structural evolutions of methane hydrate particles in aqueous solutions (PDF)
Visualization of evolutions of two methane hydrate particles with a radius of 10 nm (**Section A1**) at 275 K. All water beads, T1 beads, and T2 beads are colored cyan, red, and white, respectively. (Movie S1) (MP4)
Visualization of evolutions of two methane hydrate particles with a radius of 8 nm (**Section A2**) at 275 K. All water beads, T1 beads, and T2 beads are colored cyan, red, and white, respectively. (Movie S2) (MP4)
Visualization of evolutions of two methane hydrate particles with a radius of 6 nm (**Section A3**) at 275 K. All water beads, T1 beads, and T2 beads are colored cyan, red, and white, respectively. (Movie S3) (MP4)
Visualization of evolutions of two methane hydrate particles with a radius of 4 nm (**Section A4**) at 275 K. All water beads, T1 beads, and T2 beads are colored cyan, red, and white, respectively. (Movie S4) (MP4)
Visualization of evolutions of two methane hydrate particles with radii of 10 nm and 5 nm, respectively (**Section B1**) at 275 K. All water beads, T1 beads, and T2 beads are colored cyan, red, and white, respectively. (Movie S5) (MP4)
Visualization of evolutions of two methane hydrate particles with radii of 10 nm and 2 nm, respectively (**Section B2**) at 275 K. All water beads, T1 beads, and T2 beads are colored cyan, red, and white, respectively. (Movie S6) (MP4)
Visualization of evolutions of two ellipsoidal methane hydrate particles (**Section C1**)­at 275 K. The specific dimensions are shown in Table 3. All water beads, T1 beads, and T2 beads are colored cyan, red, and white, respectively. (Movie S7) (MP4)
Visualization of the evolution of two ellipsoidal methane hydrate particle systems (**Section C2**)­at 275 K. The specific dimensions are shown in Table 3. All water beads, T1 beads, and T2 beads are colored cyan, red, and white, respectively. (Movie S8) (MP4)
Visualization of evolutions of three methane hydrate particles with a radius of 10 nm (**Section D1**)­at 275 K. All water beads, T1 beads, and T2 beads are colored cyan, red, and white, respectively. (Movie S9) (MP4)
Visualization of evolutions of four methane hydrate particles with a radius of 10 nm (**Section D2**) at 275 K. All water beads, T1 beads, and T2 beads are colored cyan, red, and white, respectively. (Movie S10) (MP4)
