# Supplementary material for: Agglomerations of Methane Hydrate Particles in Aqueous Solutions: Insight from Dissipative Particle Dynamics Simulations
Source: ACS Omega. 2026 Mar 5;11(10):15981–93. doi: 10.1021/acsomega.5c10009 (PMC13000610; doi:10.1021/acsomega.5c10009)
Supplement: Supplementary file 1 [file ao5c10009_si_001.pdf]

Supplementary Materials for

Agglomerations of Methane Hydrate Particles in Aqueous Solutions: Insight from Dissipative Particle Dynamics Simulations

Minglei Wang, Pinqiang Cao*

School of Resource and Environmental Engineering, Wuhan University of Science and Technology, Wuhan, Hubei 430081, China

* To whom correspondence should be addressed: pinqiang2022@163.com; pinqiang@wust.edu.cn

This PDF File Includes:

Figure S1, S3, andS5. Structural evolutions of two methane hydrate particles in aqueous solutions with a radius of 10 nm, 8 nm, and 4 nm, respectively.

Figure S2, S4, and S6. Evolutions of parameter of two methane hydrate particles in aqueous solutions with a radius of 10 nm, 8 nm, and 4 nm, respectively.

Figure S7. Evolutions of parameter of two methane hydrate particles with a radius of 10 nm and 2 nm, respectively.

Figure S8. Structural evolutions of two ellipsoidal methane hydrate particles in aqueous solutions.

Figure S9. Evolutions of parameter of two ellipsoidal methane hydrate particles in aqueous solutions.

Figure S10. Structural evolutions of three spherical methane hydrate particles with a radius of 10nm in aqueous solutions.

Figure S11. Evolutions of parameter of three spherical methane hydrate particles with a radius of 10

nm in aqueous solutions.

Figure S12. The mean square displacement of water beads as a function of simulation time.

Figure S13. Local structures of methane hydrate particles in aqueous solutions.

Table S1. The detail parameters of the molecular model.

Table S2. The initial coordinate information of T1 and T2 in the single crystalline cell.

Table S3. Summary table of agglomeration results.

Table S4. Summary table of diffusion coefficients of water beads

Other Supplementary Materials:

Movie S1-S10. Visualizations of structural evolutions of methane hydrate particles in aqueous solutions.

Supporting Information Figures and Tables

Figure S1

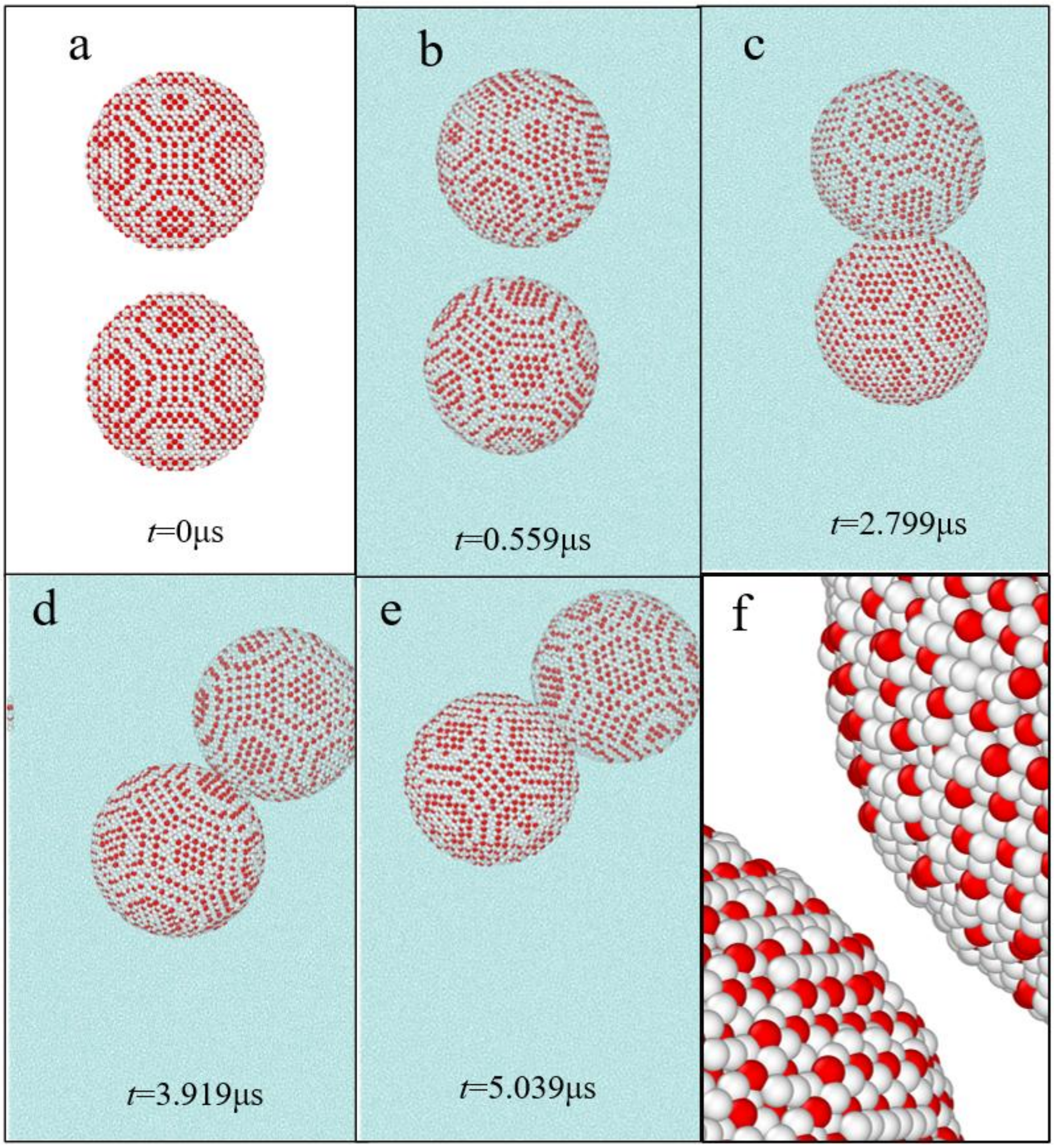

Figure S1. Structural evolutions of two methane hydrate particles with a radius of 10 nm in aqueous solutions. (a)-(e) Structural evolutions of **Section A1** in aqueous solutions at different simulation times. (f) The local structures in **Section A1** at 5.039 μs. The water beads in (a) and (f) are not shown for clarity. All water beads, T1 beads, and T2 beads are colored cyan, red, and white, respectively.

Figure S2

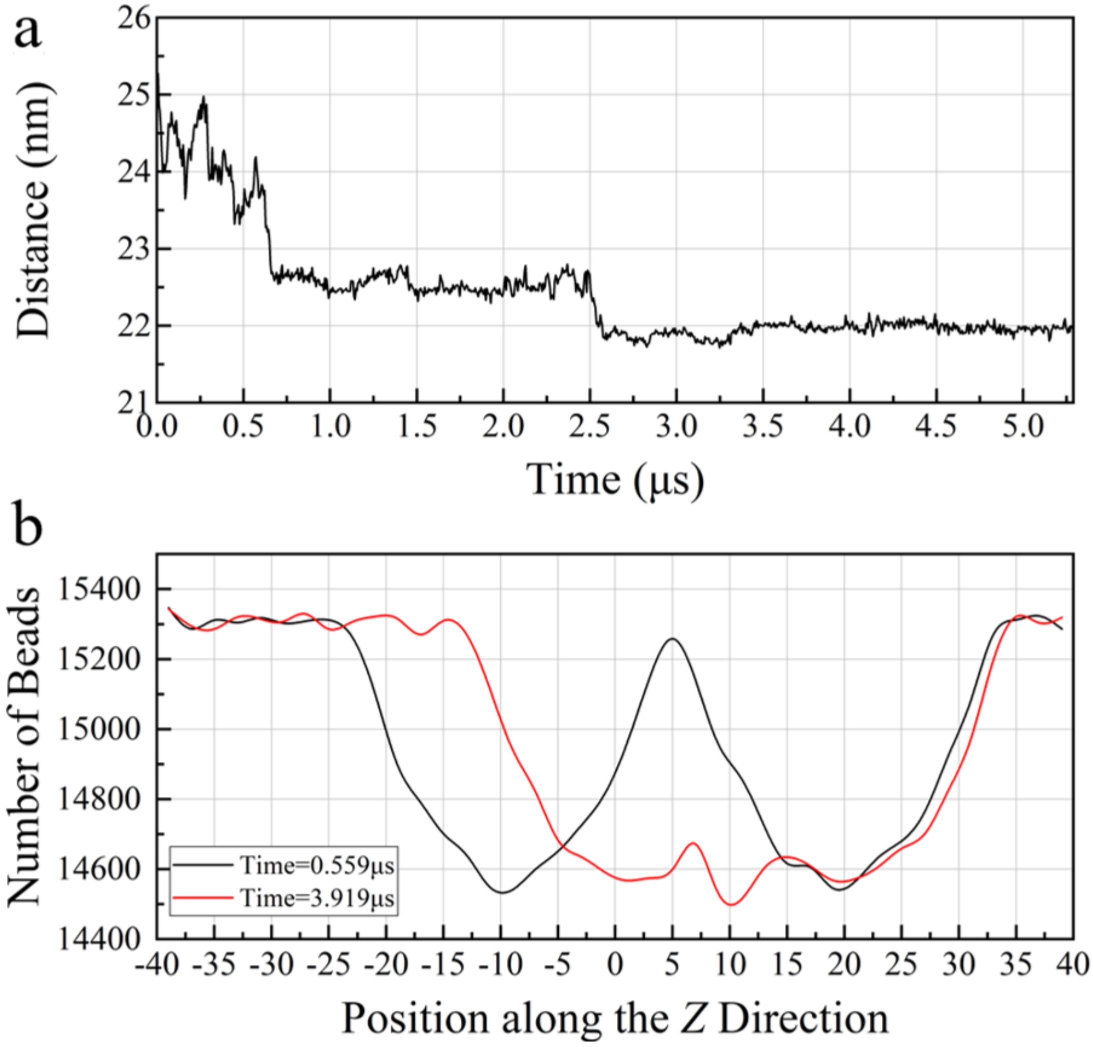

Figure S2. Evolutions of parameter of two methane hydrate particle systems with a radius of 10 nm in aqueous solutions. (a) The relative distance between methane hydrate particle centroids as a function of simulation time in **Section A1**. (b) The number of beads along the *Z* direction at different simulation times in **Section A1**.

Figure S3

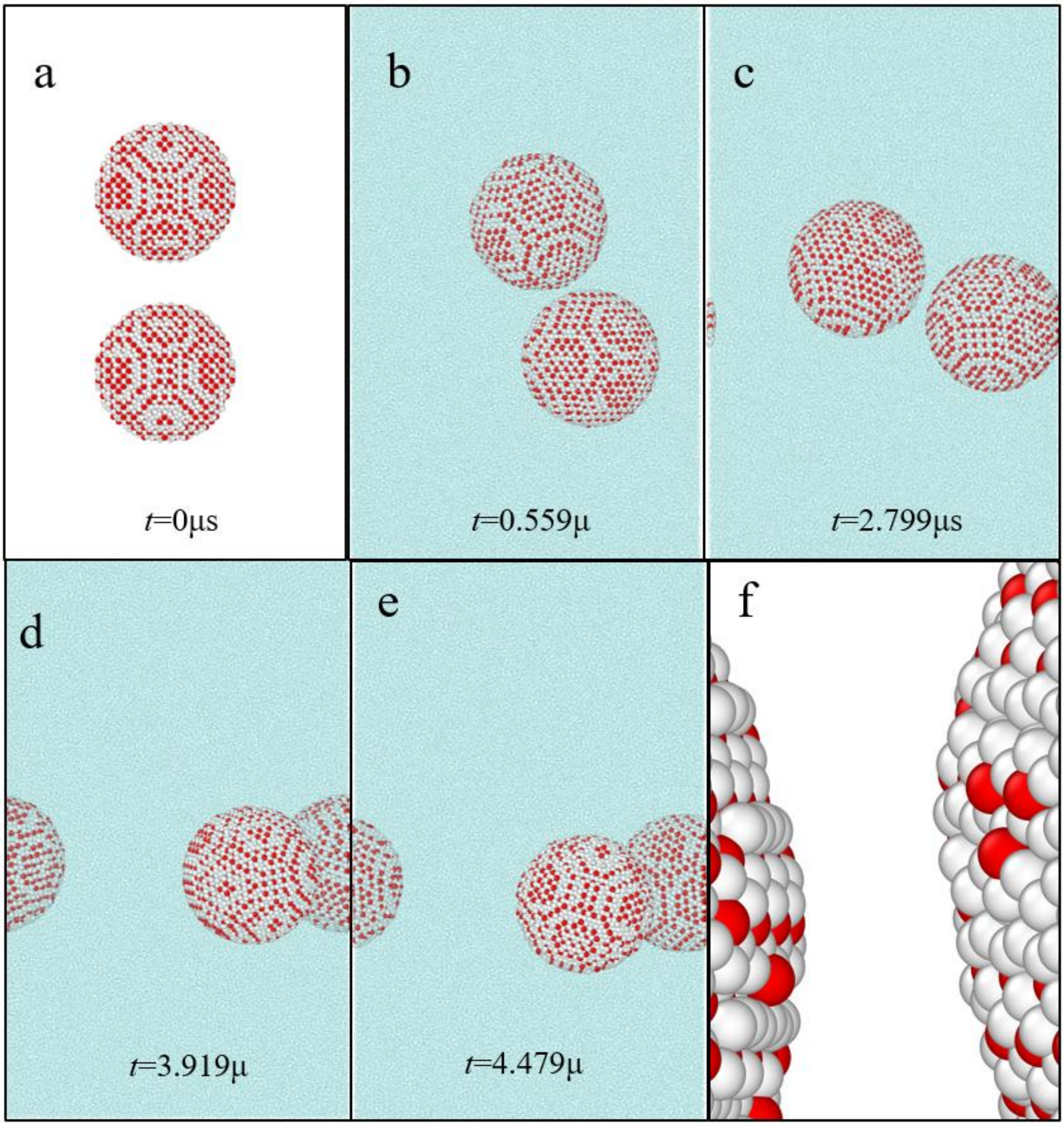

Figure S3. Structural evolutions of two methane hydrate particles with a radius of 8 nm in aqueous solutions. (a)-(e) Structures of **Section A2** in aqueous solutions at different simulation times. (f) The local structures in **Section A2** at 4.479 μs. The water beads in (a) and (f) are not shown for clarity. All water beads, T1 beads, and T2 beads are colored cyan, red, and white, respectively.

Figure S4

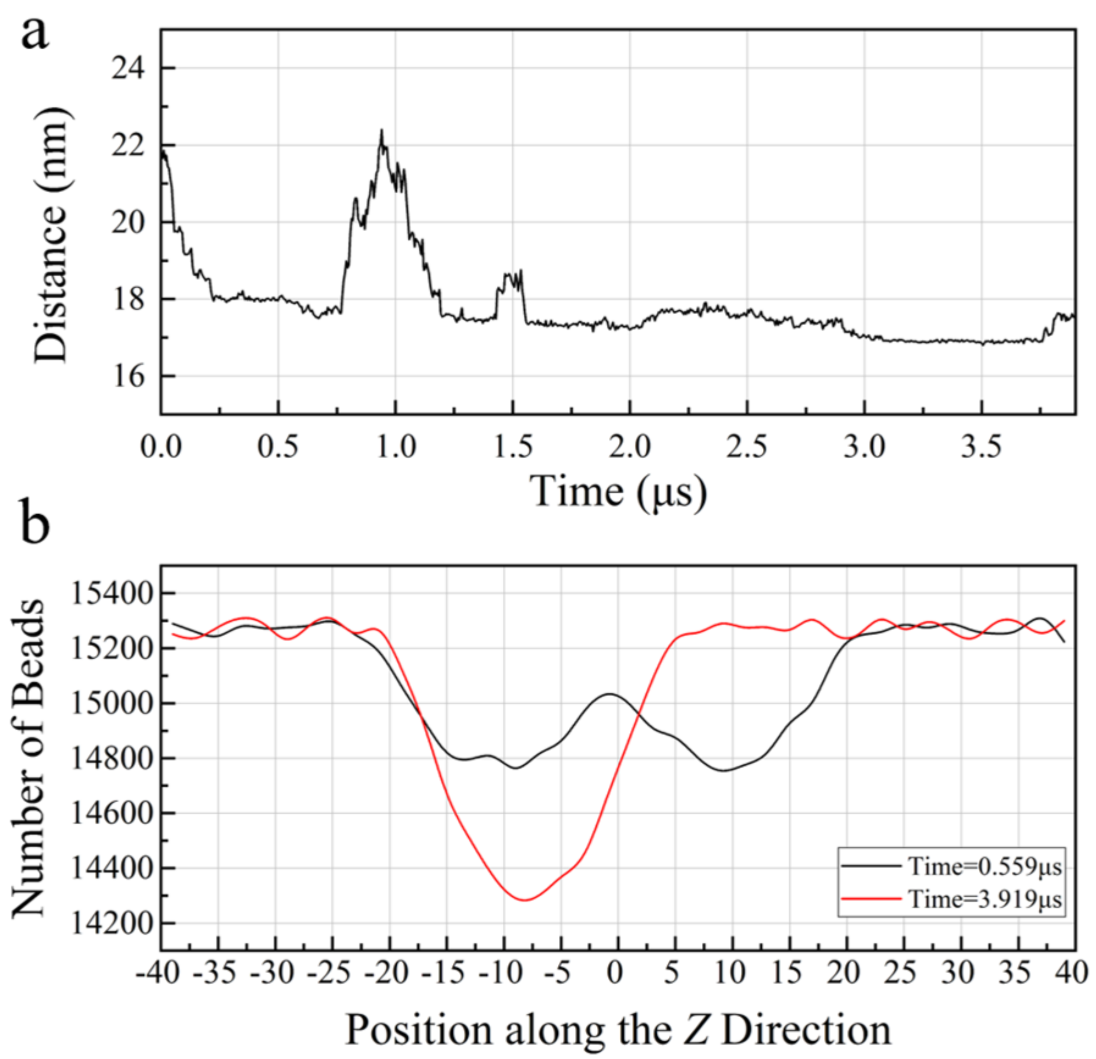

Figure S4. Evolutions of parameter of two methane hydrate particles with a radius of 8nm in aqueous solutions. (a) The relative distance between methane hydrate particle centroids as a function of simulation time in **Section A2**. (b) The number of beads along the *Z* direction at different simulation times in **Section A2**.

Figure S5

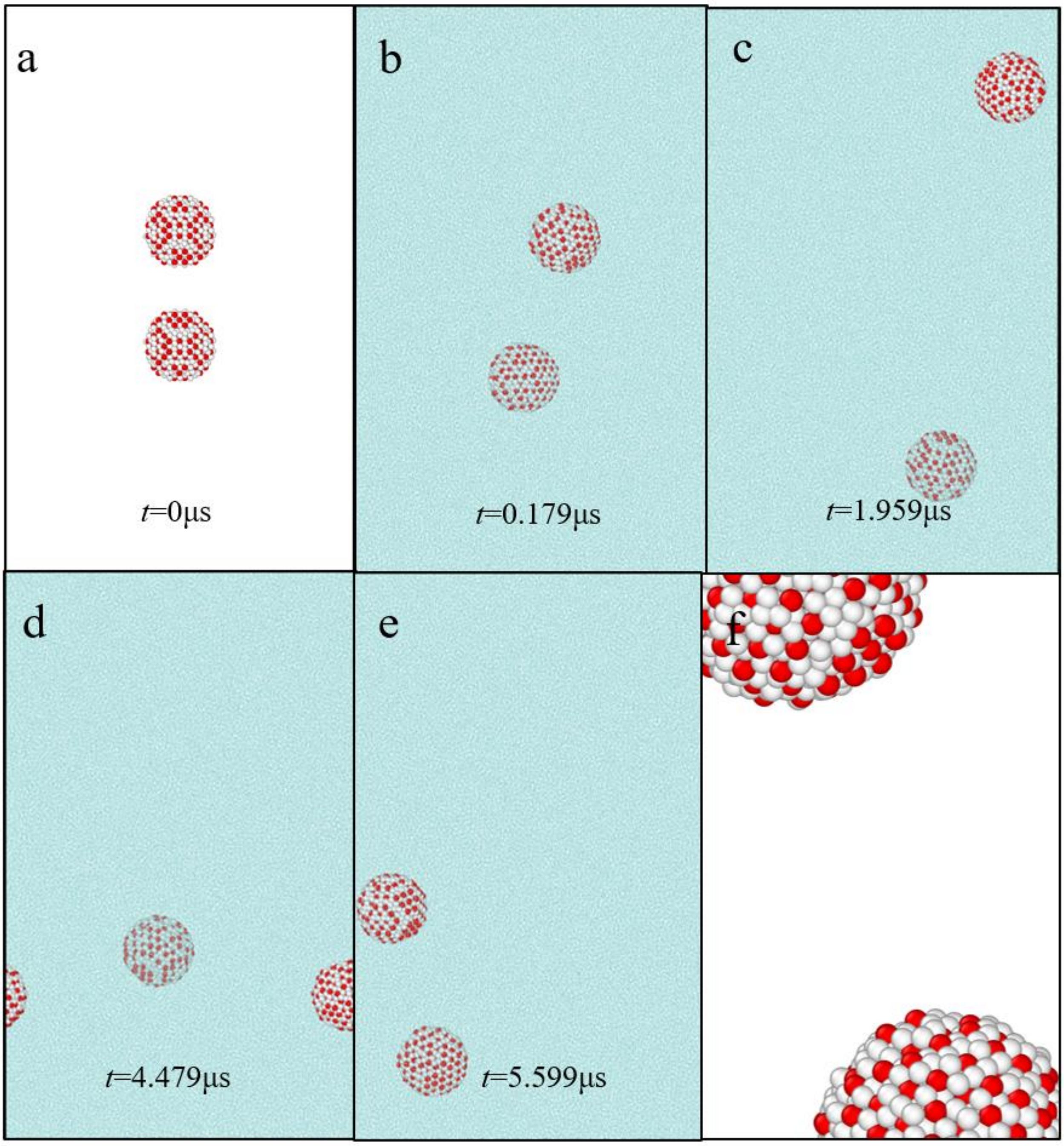

Figure S5. Structural evolutions of two methane hydrate particles with a radius of 4 nm in aqueous solutions. (a)-(e) Structures of **Section A4** in aqueous solutions at different simulation times. (f) The local structure in **Section A4** at 5.599 μs. The water beads in (a) and (f) are not shown for clarity. All water beads, T1 beads, and T2 beads are colored cyan, red, and white, respectively.

Figure S6

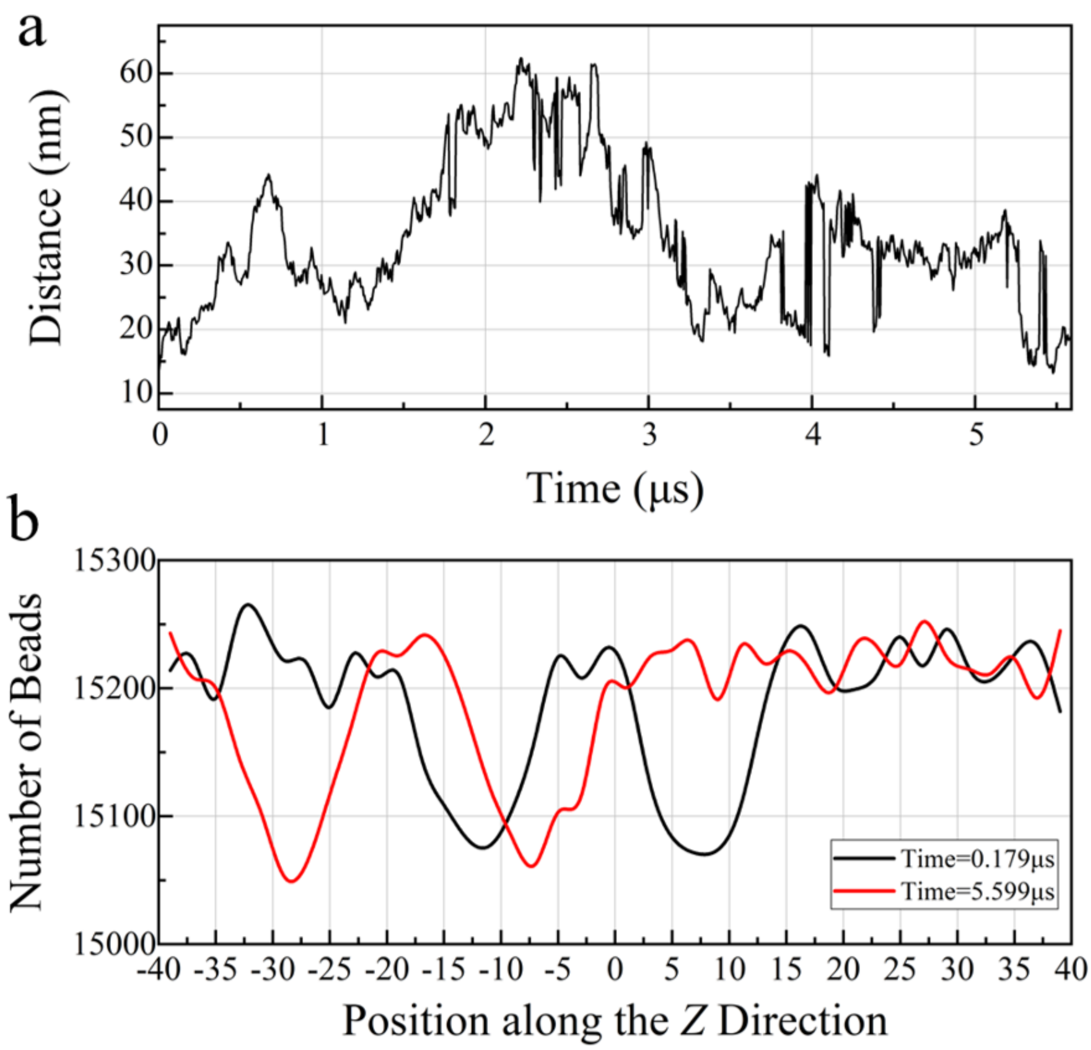

Figure S6. Evolutions of parameter of two methane hydrate particles with a radius of 4nm in aqueous solutions. (a) The relative distance between methane hydrate particle centroids as a function of simulation times in **Section A4**. (b) The number of beads along the *Z* direction at different simulation times in **Section A4**.

Figure S7

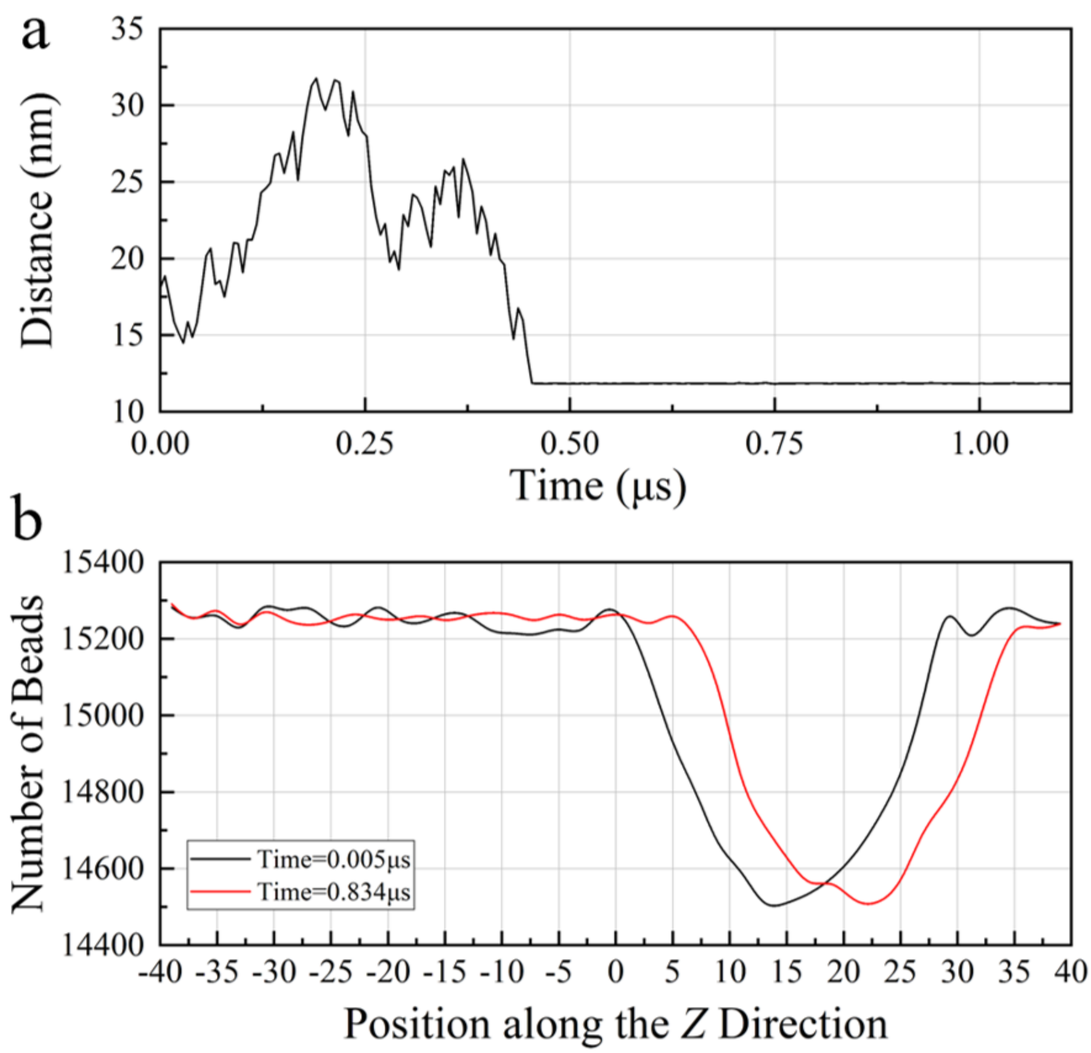

Figure S7. Evolutions of parameter of two methane hydrate particles with a particle radius of 10nm and 2 nm, respectively, in aqueous solutions. (a) The relative distance between of methane hydrate particle centroids as a function of simulation time in **Section B2**. (b) The number of beads along the *Z* direction at different simulation times in **Section B2**.

Figure S8

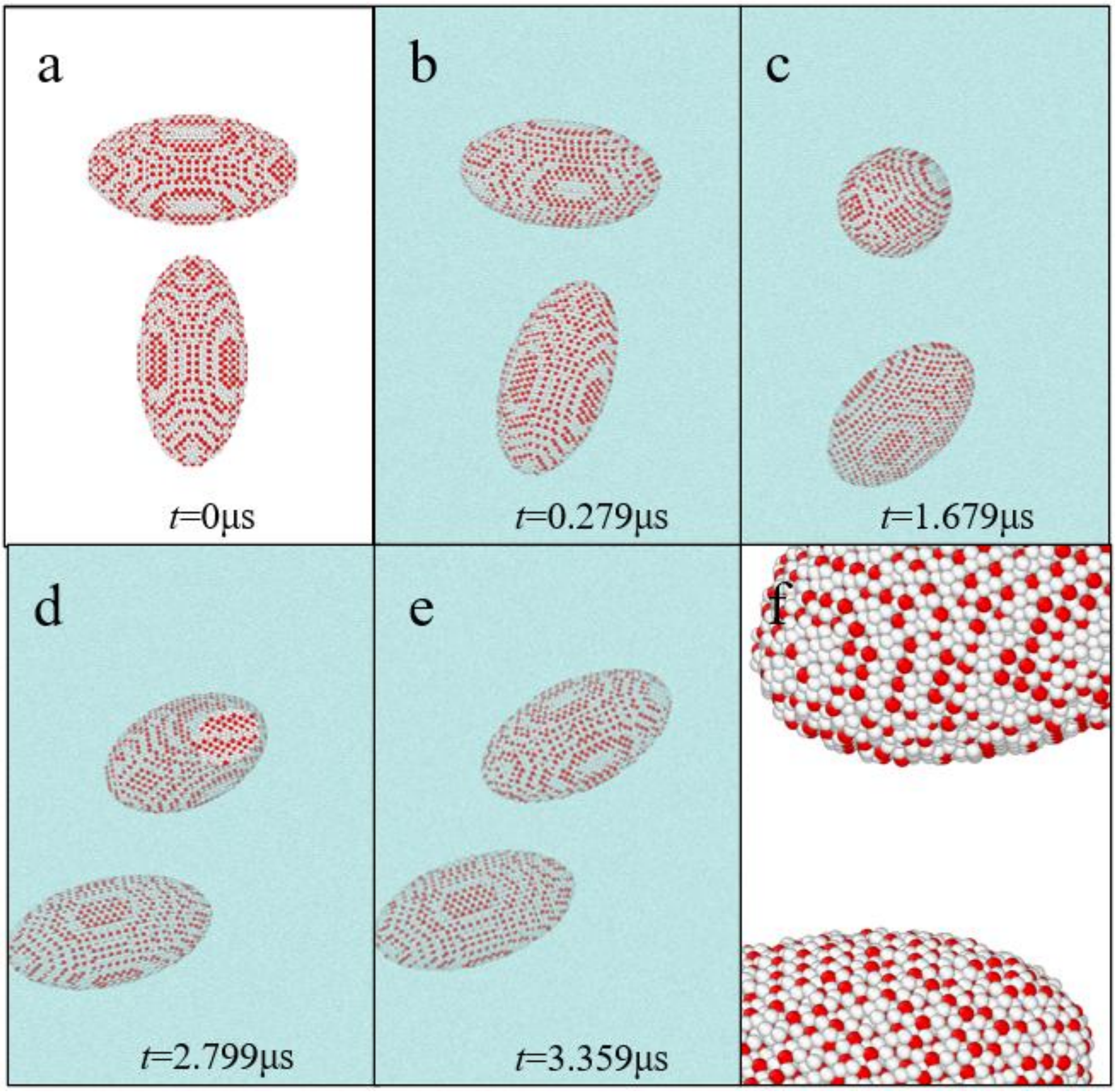

Figure S8. Structural evolutions of two ellipsoidal methane hydrate particles with dimensions of 8 nm×8 nm×15.6 nm placed vertical to each other in aqueous solutions. (a)-(e) Structures of **Section C2** at different simulation times. (f) The local structure in **Section C2** at 3.359 μs. The water beads in (a) and (f) are not shown for clarity. All water beads, T1 beads, and T2 beads are colored cyan, red, and white, respectively.

Figure S9

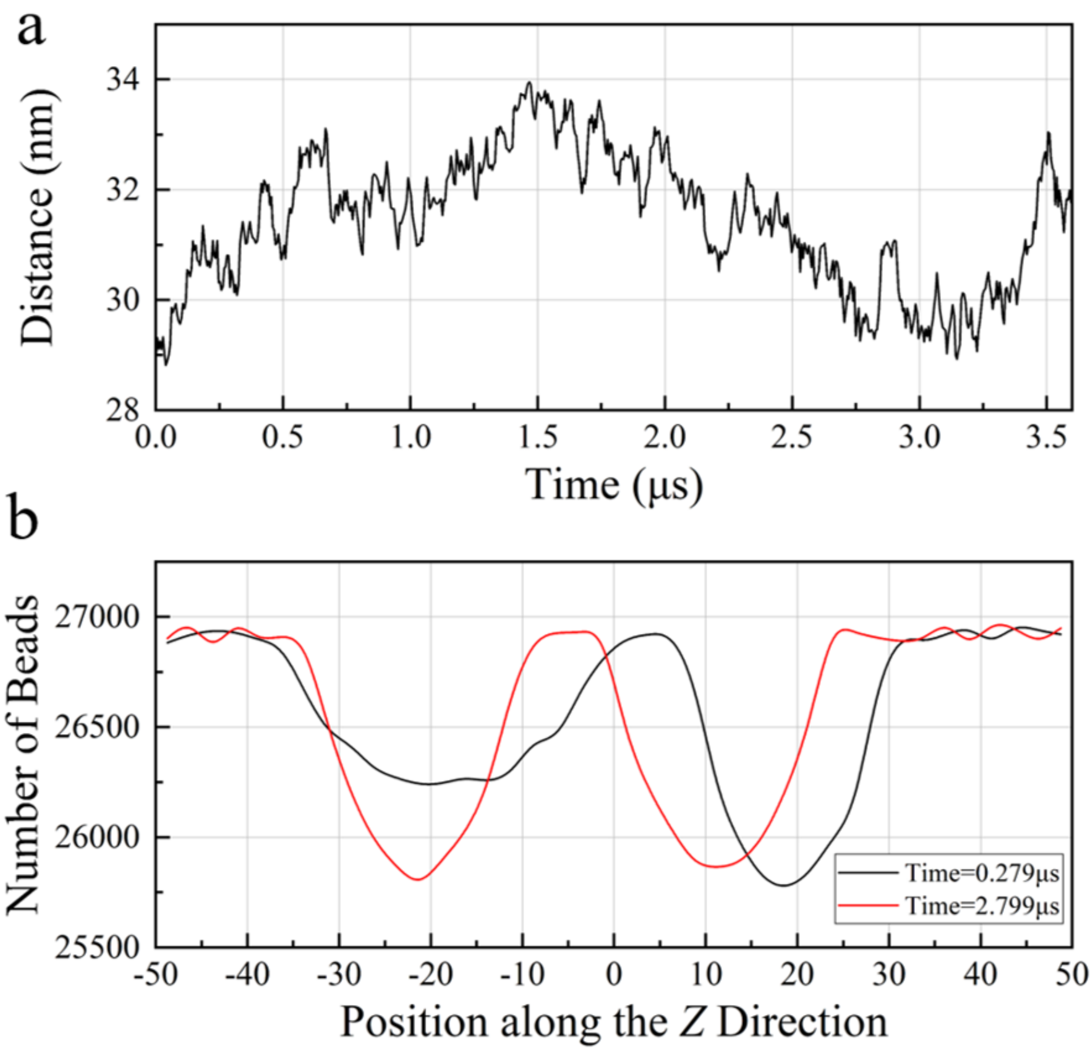

Figure S9. Evolutions of parameter of two ellipsoidal methane hydrate particles with dimensions of 8 nm×8 nm×15.6 nm placed vertical to each other in aqueous solutions. (a) The relative distance between methane hydrate particle centroids as a function of simulation time in **Section C2**. (b) The number of beads along the *Z* direction at different simulation times in **Section C2**.

Figure S10

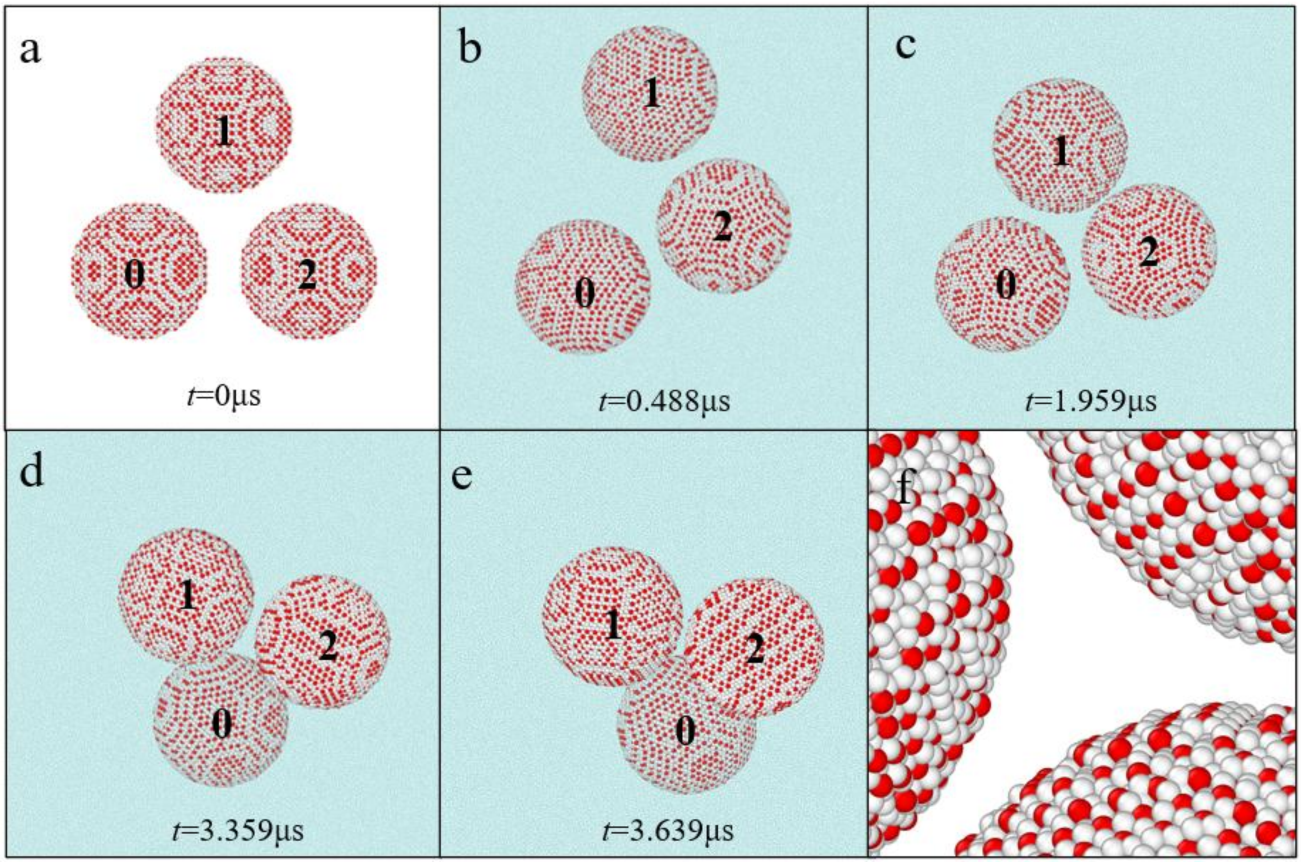

Figure S10. Structural evolutions of three spherical methane hydrate particles with a radius of 10 nm in aqueous solutions. (a)-(e) Structures of **Section D1** in aqueous solutions at different simulation times. (f) The local structure in **Section D1** at 3.639 μs. The water beads in (a) and (f) are not shown for clarity. All water beads, T1 beads, and T2 beads are colored cyan, red, and white, respectively.

Figure S11

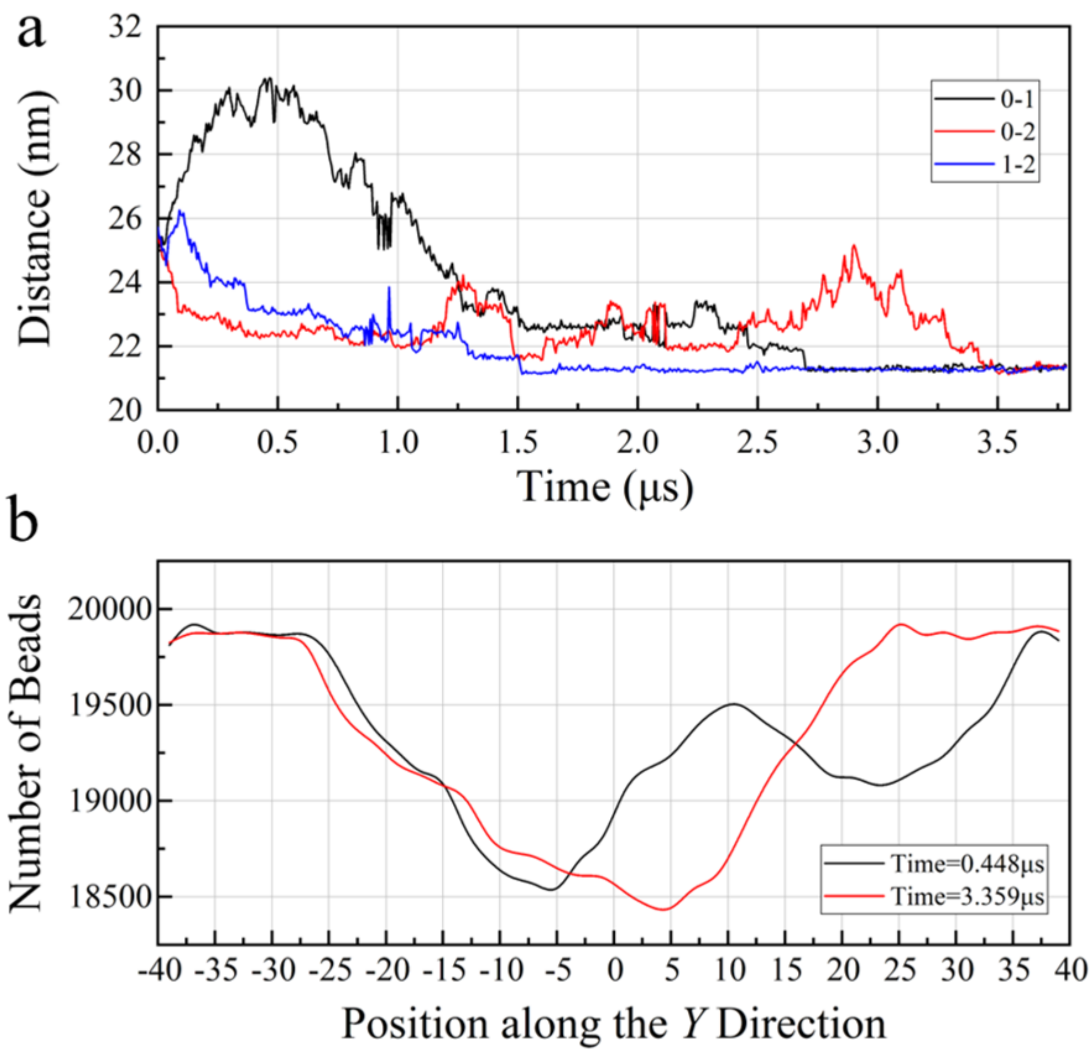

Figure S11. Evolutions of parameter of three spherical methane hydrate particle systems with radius of 10 nm in aqueous solutions. (a) The relative distance between methane hydrate particle centroids as a function of simulation time in **Section D1**. (b) The number of beads along the *Y* direction at different simulation times in **Section D1**.

Figure S12

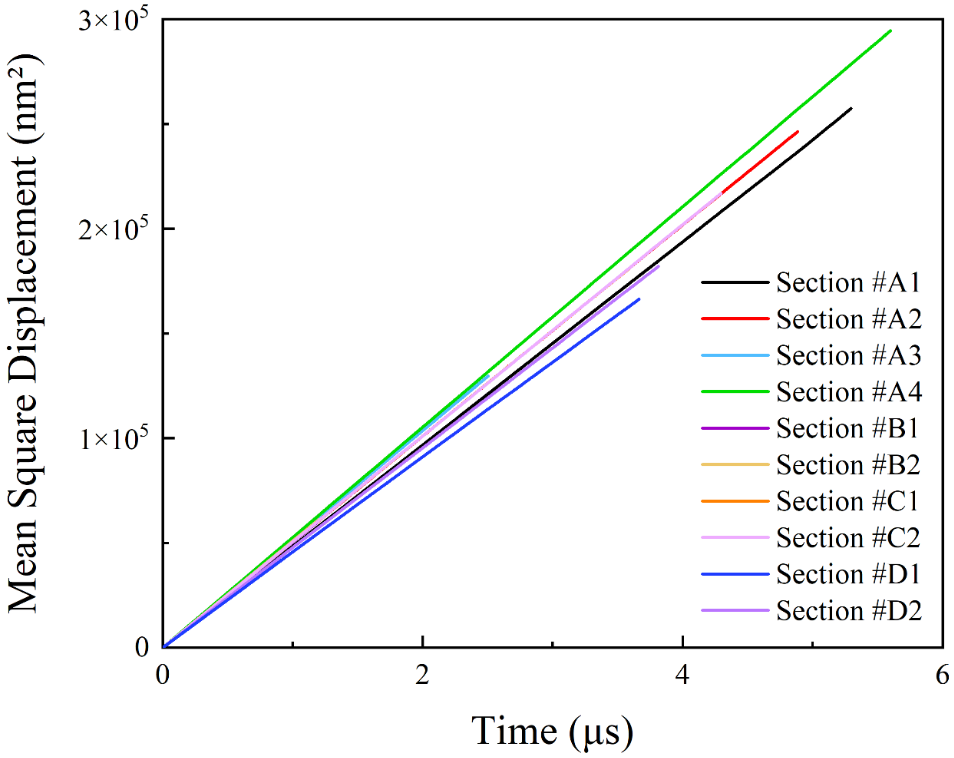

Figure S12. The mean square displacement of water beads as a function of simulation time.

Figure S13

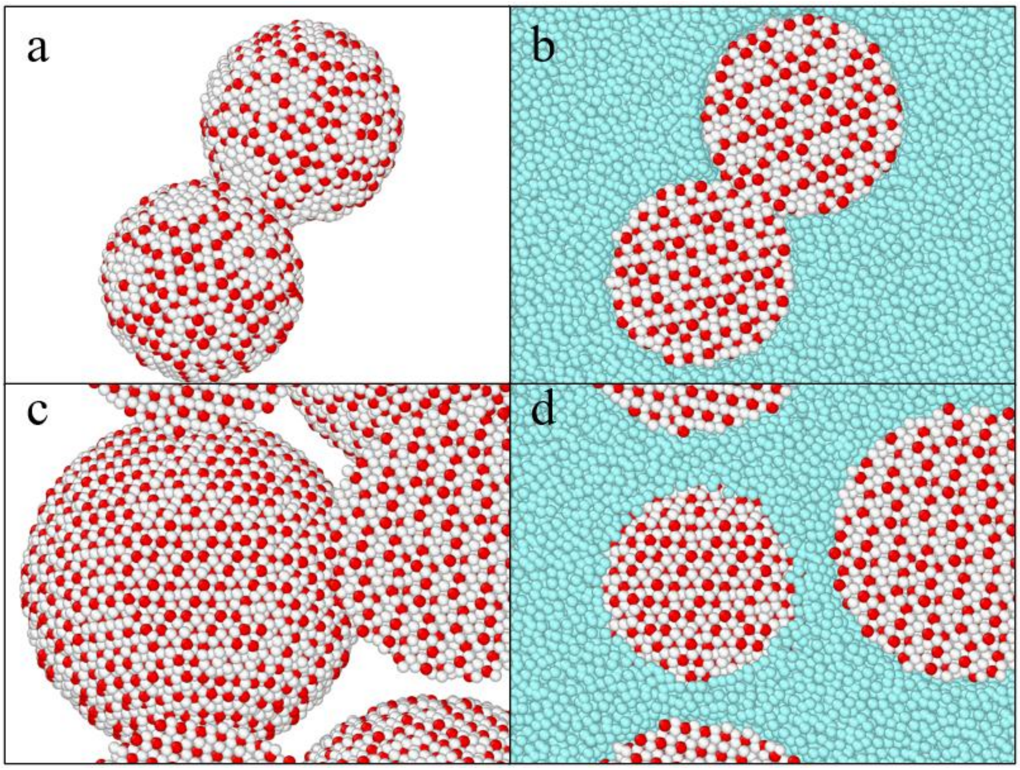

Figure S13. Local structures of methane hydrate particles in aqueous solutions. (a) Local structures of hydrate particle systems under the stable agglomeration state with direct contact in **Section A3**. (b) The cross-sectional view corresponding to (a) in **Section A3**. (c) Local structures of hydrate particle systems under the metastable agglomeration state without direct contact in **Section D2**. (d) The cross-sectional view corresponding to (c) in **Section D2**. The water beads in (a) and (c) are not shown for clarity. All water beads, T1 beads, and T2 beads are colored cyan, red, and white, respectively.

Table S1

Table S1. The detail parameters of the molecular model.

Molecular model	H_2O	CH_4
molecular weight(g/mol)	18.015	16.043
Bond length(Å)	$r_{O\text{-}H}$=0.95	$r_{C\text{-}H}$=1.14
bond Angle	$\theta_{H\text{-}O\text{-}H}$=104.45°	$\theta_{H\text{-}C\text{-}H}$=109.5°

Table S2

Table S2. The initial coordinate information of T1 and T2 in the unit cubic cell. (units: Å)

Bead type	Coordinate(Å)	Bead type	Coordinate(Å)
1-T1	(6.117411,6.105297,6.060641)	5-T2	(9.037164,6.414636,11.377757)
2-T1	(11.707968,11.629208,11.792612)	6-T2	(5.927265,11.560637,3.004865)
3-T2	(0.648910,2.941190,6.290390)	7-T2	(6.499917,11.591121,9.230811)
4-T2	(0.652074,9.185783,5.817202)	8-T2	(2.922508,5.460585,0.670564)

Table S3

Table S3. Summary table of agglomeration results. Here MASDC represents the metastable agglomeration state without direct contact, SASDC represents the stable agglomeration state with direct contact, Free represents free movements of methane hydrate particles by translation and rotation without agglomeration phenomenon observed within the simulation time.

Group	Result	Group	Result
Section A1	MASDC	Section B2	SASDC
Section A2	MASDC	Section C1	MASDC
Section A3	SASDC	Section C2	Free
Section A4	Free	Section D1	MASDC
Section B1	SASDC	Section D2	MASDC

Table S4

Table S4 Summary table of diffusion coefficients of water beads

Group	Diffusion coefficient($nm^2/\mu s$)	Group	Diffusion coefficient($nm^2/\mu s$)
Section A1	8108.56	Section B2	8485.93
Section A2	8405.69	Section C1	8405.98
Section A3	8634.99	Section C2	8426.53
Section A4	8767.47	Section D1	7955.36
Section B1	8392.08	Section D2	7564.73

Supporting Information Movies

Movies S1

Movies S1. Visualization of evolutions of two methane hydrate particles with a radius of 10 nm (**Section A1**) at 275K. All water beads, T1 beads, and T2 beads are colored cyan, red, and white, respectively.

Movies S2

Movies S2. Visualization of evolutions of two methane hydrate particles with a radius of 8 nm (**Section A2**) at 275K. All water beads, T1 beads, and T2 beads are colored cyan, red, and white, respectively.

Movies S3

Movies S3. Visualization of evolutions of two methane hydrate particles with a radius of 6 nm (**Section A3**) at 275K. All water beads, T1 beads, and T2 beads are colored cyan, red, and white, respectively.

Movies S4

Movies S4. Visualization of evolutions of two methane hydrate particles with a radius of 4 nm (**Section A4**) at 275K. All water beads, T1 beads, and T2 beads are colored cyan, red, and white, respectively.

Movies S5

Movies S5. Visualization of evolutions of two methane hydrate particles with a radius of 10nm and 5nm, respectively, (**Section B1**) at 275K. All water beads, T1 beads, and T2 beads are colored cyan, red, and white, respectively.

Movies S6

Movies S6. Visualization of evolutions of two methane hydrate particles with a radius of 10nm and 2nm, respectively, (**Section B2**) at 275K. All water beads, T1 beads, and T2 beads are colored cyan, red, and white, respectively.

Movies S7

Movies S7. Visualization of evolutions of two ellipsoidal methane hydrate particles (**Section C1**) at 275K, the specific dimensions are shown in Table 3. All water beads, T1 beads, and T2 beads are colored cyan, red, and white, respectively.

Movies S8

Movies S8. Visualization of evolutions of two ellipsoidal methane hydrate particles system (**Section C2**) at 275K, the specific dimensions are shown in Table 3. All water beads, T1 beads, and T2 beads are colored cyan, red, and white, respectively.

Movies S9

Movies S9. Visualization of evolutions of three methane hydrate particles with a radius of 10 nm (**Section D1**) at 275K. All water beads, T1 beads, and T2 beads are colored cyan, red, and white, respectively.

Movies S10

Movies S10. Visualization of evolutions of four methane hydrate particles with a radius of 10 nm (**Section D2**) at 275K. All water beads, T1 beads, and T2 beads are colored cyan, red, and white, respectively.
